# The secretome of macrophages has a differential impact on spinal cord injury recovery according to the polarization protocol

**DOI:** 10.3389/fimmu.2024.1354479

**Published:** 2024-02-20

**Authors:** José Lentilhas-Graça, Diogo J. Santos, João Afonso, Andreia Monteiro, Andreia G. Pinho, Vera M. Mendes, Marta S. Dias, Eduardo D. Gomes, Rui Lima, Luís S. Fernandes, Fernando Fernandes-Amorim, Inês M. Pereira, Nídia de Sousa, Jorge R. Cibrão, Aline M. Fernandes, Sofia C. Serra, Luís A. Rocha, Jonas Campos, Tiffany S. Pinho, Susana Monteiro, Bruno Manadas, António J. Salgado, Ramiro D. Almeida, Nuno A. Silva

**Affiliations:** ^1^ Life and Health Sciences Research Institute (ICVS), School of Medicine, University of Minho, Braga, Portugal; ^2^ ICVS/3B’s Associate Lab, PT Government Associated Lab, Braga, Portugal; ^3^ CNC—Center for Neuroscience and Cell Biology, University of Coimbra, Coimbra, Portugal; ^4^ iBiMED- Institute of Biomedicine, Department of Medical Sciences, University of Aveiro, Aveiro, Portugal

**Keywords:** spinal cord injury, macrophages, secretome, neuroimmunology, neuroregeneration

## Abstract

**Introduction:**

The inflammatory response after spinal cord injury (SCI) is an important contributor to secondary damage. Infiltrating macrophages can acquire a spectrum of activation states, however, the microenvironment at the SCI site favors macrophage polarization into a pro-inflammatory phenotype, which is one of the reasons why macrophage transplantation has failed.

**Methods:**

In this study, we investigated the therapeutic potential of the macrophage secretome for SCI recovery. We investigated the effect of the secretome in vitro using peripheral and CNS-derived neurons and human neural stem cells. Moreover, we perform a pre-clinical trial using a SCI compression mice model and analyzed the recovery of motor, sensory and autonomic functions. Instead of transplanting the cells, we injected the paracrine factors and extracellular vesicles that they secrete, avoiding the loss of the phenotype of the transplanted cells due to local environmental cues.

**Results:**

We demonstrated that different macrophage phenotypes have a distinct effect on neuronal growth and survival, namely, the alternative activation with IL-10 and TGF-β1 (M(IL-10+TGF-β1)) promotes significant axonal regeneration. We also observed that systemic injection of soluble factors and extracellular vesicles derived from M(IL-10+TGF-β1) macrophages promotes significant functional recovery after compressive SCI and leads to higher survival of spinal cord neurons. Additionally, the M(IL-10+TGF-β1) secretome supported the recovery of bladder function and decreased microglial activation, astrogliosis and fibrotic scar in the spinal cord. Proteomic analysis of the M(IL-10+TGF-β1)-derived secretome identified clusters of proteins involved in axon extension, dendritic spine maintenance, cell polarity establishment, and regulation of astrocytic activation.

**Discussion:**

Overall, our results demonstrated that macrophages-derived soluble factors and extracellular vesicles might be a promising therapy for SCI with possible clinical applications.

## Background

Spinal cord injury (SCI) is a devastating neurological disorder that strongly affects the physiological, psychological, and social behaviors of affected people. There is an urgent need to develop new therapeutic strategies for SCI repair (1). The spinal cord trauma, known as “primary injury”, triggers a cascade of events, termed “secondary injury”, leading to further neurological damage and contributing to regeneration failure after SCI (2). These include glutamate excitotoxicity, a potent and dysfunctional inflammatory response, release of molecules that inhibit axonal growth, and formation of a glial scar. From all these events, the defective immune response is one of the most important players in SCI pathophysiology. Circulating monocytes infiltrate the spinal cord and differentiate into macrophages in a multiphasic manner, where they should perform multiple functions involved in the wound healing process (3). It was recently demonstrated that the spleen releases the first monocytes that infiltrate the injured spinal cord (4). Moreover, Swirsky et al. characterized the splenic monocyte reservoir as a major source of the pro-inflammatory subtype during acute injury (5).

Macrophages can acquire a diverse spectrum of activation states with various functionalities. Macrophage activation can range from the most pro-inflammatory or classically activated phenotype to the anti-inflammatory/pro-repair or alternatively activated phenotype. Pro-inflammatory macrophages are important during the acute response to trauma and facilitate innate immunity to remove wound debris from the injury site. These macrophages release reactive oxygen species (ROS) and pro-inflammatory cytokines, such as IL-1β and TNF-α (6). Macrophages can acquire this phenotype *in vitro* by stimulating naïve macrophages with lipopolysaccharide (LPS) and IFN-γ (commonly known as M1). In contrast, alternatively activated macrophages secrete immunosuppressive cytokines, growth factors, and upregulate ECM components (e.g., IL-10, TGF-β1, and IGF-1) (7, 8). These macrophages exhibit tissue repair properties by promoting cell proliferation and maturation, tissue remodeling and stabilization, and adjusting and resolving inflammatory processes. These tasks are not performed by a single type of alternatively activated macrophage. Instead, they are subdivided into four distinct subtypes (commonly known as M2a, M2b, M2c, and M2d) that differ in cell surface markers, secreted cytokines, and biological functions (6). Herein, we focus on two alternatively activated macrophages, the M2a and M2c. The first can be obtained *in vitro* by stimulating naïve macrophages with IL-4 and IL-13, and their function is associated with a decrease in the inflammatory response, promotion of cell proliferation and migration, and facilitation of apoptosis. After SCI these cells fail to activate an appropriate pro-regenerative response (6). Whereas, the M2c macrophages have functions related to resolving inflammation, ECM synthesis, and promoting tissue maturation/repair. These cells can be obtained by activating naïve macrophages with TGF-β1 and IL-10. The significance of M2c cells in SCI repair remains largely unexplored because these cells do not populate the lesion site, impeding the initiation of the remodeling phase (6). Overall, the immune response at the initial stages after SCI resembles that in non-CNS injured tissues (9). However, pro-inflammatory macrophages quickly become the predominant cell type at the injury site (10), and pro-repair macrophages are unable to populate the injured tissue. The pro-inflammatory response is associated with fibrosis, oxidative damage, and neurodegeneration, contributing to wound healing failure (11).

Previous studies transplanted alternatively activated macrophages into the injured spinal cord to promote tissue repair and regeneration (12, 13). This therapeutic approach reached clinical testing, but failed to show any therapeutic effects (14). The reason behind this clinical trial failure may lie in the spinal cord microenvironment after injury. Indeed, a previous study reported that bone marrow-derived macrophages polarized *in vitro* by IL-4 failed to retain their typical markers when transplanted into the injured spinal cord (10). Moreover, it was demonstrated that intracellular accumulation of iron by macrophages induces a rapid switch from a pro-regenerative to a pro-inflammatory phenotype in spinal cord tissue (15). Thus, it is important to find alternative approaches for M2 macrophage transplantation. A possible alternative is to administer the secretome of macrophages instead of transplanting them into the SCI microenvironment. The secretome can be defined as the soluble factors, lipids, and extracellular vesicles secreted by a cell, tissue, or organism into the extracellular space under defined time and conditions (16).

Herein, we explored whether systemic injections of secretome derived from different macrophage phenotypes have a therapeutic effect after SCI.

## Materials and methods

### Macrophages isolation and culture

Macrophages were obtained by differentiating monocytes extracted from the mouse spleens. C57BL/6 mice (~8 weeks old) were sacrificed by cervical dislocation, and their spleen was removed under aseptic conditions and kept on ice-cold VLE-RPMI 1640 (Merck KGaA) with 1% (v/v) penicillin-streptomycin (pen/strep, Gibco). The spleen was mechanically dissociated using two microscope slides until no major fragments were observed. The solution was centrifuged at 1200 rpm for 7 min and the supernatant was discarded. Ammonium-chloride-potassium (ACK) lysis solution was used to lyse erythrocytes (2mL/spleen). After adding HBSS (8mL/spleen, Gibco), centrifugation was performed, and the cell pellet was resuspended in RPMI for hematocytomer cell counting. Cells were plated at a density of 1 million cells/cm^2^ in RPMI medium 1% (v/v) pen/strep (Gibco) for 3 h. The monocytes (≈10% of the total cells) are the first to adhere under serum starvation. After this time, the non-adherent cells were discarded and the medium was replaced by RPMI with 10% (v/v) fetal bovine serum (FBS, Millipore), 1% (v/v) pen/strep, and 50 ng/mL of macrophage colony-stimulating factor (M-CSF, Biolegend) to differentiate monocytes into macrophages. The cells were maintained at 37°C and 5% (v/v) CO_2_ for a minimum of 7 days, with medium exchanges every 3/4 days. To achieve a pro-inflammatory phenotype, macrophages were stimulated with IFN-γ (20 ng/mL, Peprotech) and LPS (100 ng/mL, Sigma) for 24 h. One pro-regenerative phenotype was achieved by stimulation with IL-4 (20 ng/mL, Biolegend) and IL-13 (20 ng/mL, Peprotech), and the other phenotype was obtained with IL-10 (20 ng/mL, Peprotech) and TGF-β1 (20ng/mL, R&D Systems) stimulation. All polarizations were performed in RPMI with 10% FBS, 1% (v/v) pen/strep and 50 ng/mL of M-CSF.

The macrophage secretome was collected after each polarization. Briefly, cells were washed five times with PBS without Ca^2+^ and Mg^2+^ (Merck, KGaA), followed by two washes with RPMI 1% (v/v) pen/strep. After a 12-hour incubation with 16 mL (213ul/cm^2^) of basal medium (RPMI) with 1% (v/v) pen/strep, the medium was collected, centrifuged at 1200 rpm for 5 min, and the supernatant was snap frozen with liquid nitrogen and stored at -80°C.

### qPCR

Macrophage mRNA levels were analyzed using qPCR by extracting RNA from cells grown in T25 flasks. Briefly, 6 h after polarization, TripleXtractor (Grisp) was added to the flasks for 5 min. RNA was extracted and diluted in GRS PCR Grade Water (Grisp) following the manufacturer’s instructions. cDNA was synthesized from 1 µg of RNA using the Xpert cDNA Synthesis Supermix (with gDNA eraser, Grisp) protocol. qPCR was performed on these samples using Xpert Fast SYBR blue mastermix (Grisp) with ROX reference dye. After mixing the mastermix with the respective primers (500 nM) and the cDNA on a PCR plate (Nerbe Plus), the reaction was performed on a 7500 Fast Real-Time PCR system (Applied Biosystems). The amplification was performed by heating at 95°C for 2 minutes succeeded by 40 cycles at 95°C for 5 s and 30 s at 60°C. Melt curve analysis was used to assess the specificity of the gene amplification. The primers used are listed in **Table 1**. The target genes were normalized to three reference genes: Gadph, Hprt and 18s. Fold-change levels were calculated using the 2-ΔΔct method relative to non-stimulated macrophages and normalized to the reference genes (17).

**Table 1:** Primers for semi-quantitative Real Time-PCR.

Gene	Forward	Reverse
*GAPDH*	GGG CCC ACT TGA AGG GTG GA	TGG ACT GTG GTC ATG AGC CCT T
*HPRT*	GCT GGT GAA AAG GAC CTC T	CAC AGG ACT AGA ACA CCT GC
*18s*	GTA ACC CGT TGA ACC CCA TT	CCA TCC AAT CGG TAG TAG CG
*iNOS*	CTC GGA GGT TCA CCT CAC TGT	GCT GGA AGC CAC TGA CAC TT
*TNF-α*	GCC ACC ACG CTC TTC TGT CT	TGA GGG TCT GGG CCA TAG AAC
*EGR2*	TTG ACC AGA TGA ACG GAG TG	CCA GAG AGG AGG TGG AAG TG
*IRF4*	ACA GGA GCT GGA GGG ATT ATG	CTG TCA CCT GGC AAC CAT TT
*ARG1*	GTG TAC ATT GGC TTG CGA GA	GGT CTC TTC CAT CAC CTT GC
*HIF1-α*	GCA CTA GAC AAA GTT CAC CTG AGA	CGC TAT CCA CAT CAA AGC AA

### Axonal growth assay – dorsal root ganglia

Dorsal root ganglia (DRG) explants were used to study the impact of splenic macrophages on axonal growth. This assay was accomplished following a well-established protocol (18, 19). Briefly, DRG from thoracic regions of neonatal Wistar Han rat pups (P5-7) were removed and placed on ice-cold HBSS with 1% (v/v) pen/strep. Peripheral nerves attached to the DRG were removed, and the cleaned DRG were used. Two assays were performed. The first consisted of placing the DRG on top of a collagen extracellular matrix gel (3D culture), which was on top of polarized macrophages. Collagen gels were prepared by combining rat tail collagen type I (Corning) at a final concentration of 89.6% (v/v) with 10% (v/v) Dulbecco Modified Eagle Medium (DMEM, Gibco) 10x and 0.4% (v/v) of sodium bicarbonate (7.5% (w/v), Sigma). After forming 30 uL gel droplets at 37°C and 5% (v/v) CO_2_ for a minimum of 90 min, the gels were transferred to the macrophages’ wells. The other assay consisted of direct placement of the DRG on top of polarized macrophages to study direct cellular interactions (2D culture). Both assays were performed in Neurobasal (Gibco) medium supplemented with 2% (v/v) B27 (Gibco), 2 mM L-glutamine (Invitrogen), 6 mg/mL D-glucose (Sigma), 1% (v/v) pen/strep, and 50 ng/mL of M-CSF with medium changes every two days and maintained at 37°C and 5% (v/v) CO_2_ for four (3D) or three (2D) days. The cells were then fixed and immunocytochemistry was performed. The area occupied by the axons in each dorsal root ganglia explant was calculated using the ImageJ (NIH) plugin Neurite-J. Using confocal microscopy, the entire area with positive staining for Neurofilament was acquired. Then, the image was automatically translated to 8 bits and a binary mask was created with the aid of the “Analysis Particles” function which enables the correct segmentation of axonal structures based on an intensity-threshold image coupled with morphological parameters such as structure size and area. The mask generated can then be added as an input to the Neurite-J plugin.

### Axonal growth assay –CNS-derived neuronal culture

Cortical neurons were dissected and isolated from Wistar rats E17 embryos as described previously (20). To physically and fluidically separate distal axons from cell bodies, neurons were plated in microfluidic chambers as described previously (21). Microfluidic chambers were assembled onto an ibiTreat low wall 50 mm µ-Dish (ibidi) and coated with poly-D-lysine (PDL) 0.1 mg/mL overnight at 37°C and 2 µg/mL laminin for 2 h at 37°C. Cortical neurons were plated in the somal compartment of microfluidic chambers at a density of 50,000 cells per chamber. Cells were maintained in a humidified 5% CO_2_ incubator at 37°C and treated with 10 µM 5-Fluoro-2′-deoxyuridine (5’-FDU) on day 4 to inhibit glial cell proliferation.

On day 5, distal axons were submitted to a 20-hour starving and after which axons were treated with M_(IL-10+TGF-β1)_-derived secretome or control medium. 25 µl of secretome was locally applied to the axonal compartment of the microfluidic chamber for 14 h. Neurobasal medium with 1% penicillin/streptomycin was used for control cultures. A higher volume of culture medium was maintained in the somal compartment to ensure fluidic isolation of the axonal compartment and, therefore, restrict the treatment to distal axons. After 14 h of local treatment, population-wide axonal growth was assessed by live-cell imaging microscopy.

### Neurospheres derived from human induced neural stem cells

Neurospheres were generated by culturing human induced pluripotent stem cells (hiPSCs) *in vitro*nectin XF™ treated plates with mTeSR 1 (both from Stem Cell Technology). After 7 days, spontaneous differentiation was initiated by the generation of Embryoid Bodies (EBs). For that, cells were detached by using TrypLE™ Express Enzyme (ThermoFisher) and plate into low attachment 96 well plate in Advanced DMEM/12 supplemented with 15% (v/v) knockout serum replacement (KSR, ThermoFischer), 1% (v/v) non-essential amino acids (NEAA, ThermoFischer), 2% (v/v) glutamax (ThermoFischer), 2-mercaptoethanol (55 mM, ThermoFischer), and Y-27632 (5 mM, Rho-associated protein kinase inhibitor, StemCell Technology). The hole medium was changed every other day. On day 6, 6-9 EBs were transferred from 96 well plates to non-adherent plates (35 mm) and were cultured in Advanced DMEM/12 supplemented with 1% (v/v) non-essential amino acids, 1% (v/v) glutamax, 1% (v/v) of N2 supplement (ThermoFischer), and heparin (1μg/mL, Sigma-Aldrich) to induce neural differentiation. After 5 days, 6-9 neurospheres were plated into 24 well plates, pre-treated with poly-D-lysin/laminin (76 μg/mL, 20 μg/mL, respectively), and cultured in differentiation media: DMEM/F12: Neurobasal (1:1, both from ThermoFisher), 0.5% of N2 supplement, 1% (v/v) NEAA, 1% (v/v) glutamax, 55 mM 2-mercaptoethanol, 2% (v/v) B27 supplement (ThermoFischer), and insulin (2.5 μg/mL, Sigma). After 2 days, the culture medium was replaced by 500 µl of secretome. Cells were incubated for 2 days and fixed for further analysis using immunofluorescence.

### Immunocytochemistry

Cells/DRG/Neurospheres were first incubated with 4% (v/v) PFA for 20 min, and then permeabilized with Triton-X100 0.2% diluted in PBS (PBS-T) for 5 minutes, at room temperature (RT). 10% FBS (Millipore) in PBS was used as a blocking solution for 1 h, followed by the addition of the primary antibodies for 2 h. For macrophages was used the rat anti-CD11b (1:100, BioLegend) and rabbit anti-iNOS (1:100, Abcam), for DRGS the mouse anti-neurofilament (1:200, Millipore) and for neurospheres the Anti-βIII Tubulin (1:100, mouse – Millipore). After washing, Alexa Fluor 488 goat anti-rat (1:1000, Invitrogen) and Alexa Fluor 594 goat anti-rabbit (1:1000, Invitrogen) secondary antibodies were added for another hour, diluted in blocking solution. Finally, the samples were counterstained with 40,6-diamidino-2-phenylindoledihydrochloride (DAPI) (1 µg/mL, Sigma) for 10 min and in the case of DRGs with and Phalloidin (1:500, Sigma) for 45 min at RT. Images were obtained using a confocal microscope (Olympus FV1000) for 3D cultures and an Olympus IX81 fluorescence microscope for 2D cultures. To calculate the axonal area, maximum distance reached by axons, and axonal arborization, ImageJ software was used, as previously described (22).

### Live imaging of CNS-derived neurons

Live imaging was performed using a Zeiss LSM 880 microscope with an Airyscan and a Plan-Apo Chromat 20x/0.8 Ph2 objective. During live imaging cells were maintained in a 37°C and 5% CO_2_ environment. A tiled phase-contrast image was obtained for each condition immediately before treatment (t=0 h) and after 6, 10 and 14 hours of treatment.

Images were processed and quantified using ImageJ software version 1.51n. A region of interest (ROI) was chosen to encompass the entire length of the axonal compartment, and the same size ROI was used for all samples. The Feature J Hessian plugin was applied with the following settings: largest eigenvalue of the Hessian Tensor, smoothing scale = 2.0). The Local Threshold was adjusted to include all axons in the axonal network. A binary image was generated, and the Skeletonize (2D/3D) plugin was used to obtain a skeletonized image of the axonal network. Finally, the Analyze Skeleton (2D/3D) was applied with the following settings: prune cycle method=none, show detailed info. A Branch Information table was generated using the software, and the sum of all branch lengths was further calculated, giving the population-wide total axonal length. The results were normalized for t=0 under the respective treatment conditions.

### Spinal cord injury surgery

All experiments were performed after obtaining consent from the ethical Subcommittee in Life and Health Sciences (SECVS; ID:018/2019, University of Minho) and were conducted in accordance with the local regulations on animal care and experimentation (European Union Directive 2010/63/EU). The ARRIVE guidelines for reporting animal research have been followed (23). C57BL/6J mice (Charles River) were maintained under sterile conditions and in light, humidity, and temperature-controlled rooms. Food and water were provided *ad libitum*. Animals were handled for 1 week prior to SCI surgery.

Spinal cord surgery was performed as previously described (24). Briefly, 42 C57BL/6J adult female mice (10-15 weeks age) were used in this study. Anesthesia was delivered intraperitoneally (ip) using Imalgene (ketamine, 75 mg/kg, Richter Pharma AG) and Dormitor (medetomidine, 1 mg/kg, Pfizer). Mice were shaved and disinfected with chlorohexidine. A dorsal midline incision was then made at the thoracic level (T5-T12). The paravertebral muscles were retracted, and the spinal process and laminar arc of T8-T9 were removed to expose the spinal cord. The spinal cord was compressed using fine forceps for 5 seconds. The wound was closed with 9 mm autoclip (Braintree Scientific), and anesthesia was reverted with Antisedan (atipamezole, Orion Corporation) applied subcutaneously. The injured animals were randomly divided into four experimental groups: 1) M_(INF-γ+LPS)_ secretome (n=10); 2) M_(IL-4+IL-13)_ secretome (n=11), 3) M_(IL-10+TGF-β1)_ secretome (n=10), and 4) vehicle (RPMI medium with 1% pen/strep, n=11). Treatment was delivered by intraperitoneal injections (500 µl), and the first injections were administered 3, 6, 9, 14 days post-injury and once a week afterwards. Eight animals did not survive the experimental protocol.

In a separate cohort of animals, we employed the same protocol to induce spinal cord injury, and the same method and schedule to deliver the treatment. However, this time, we utilized (C57BL/6J x CBA)F1 mice expressing the Thy1-GFP transgene. Following spinal cord compression, the injured animals were randomly assigned to two experimental groups: 1) M_(IL-10+TGF-β1)_ secretome (n=3), and 2) vehicle (RPMI medium with 1% pen/strep, n=3). Treatment was delivered by intraperitoneal injections (500 µl) as described above.

### Post-operative care

After surgery and throughout all *in vivo* experiments, animals were closely monitored and cared for, as previously described (16). A solution containing the antibiotic enrofloxacin (Baytril, 5 mg/mL, Bayer), the analgesic buprenorphine (Bupaq, 0.05 mg/kg, Richer Pharma AG), vitamins (Duphalyte, Pfizer), and saline (0.09% NaCl) was administered subcutaneously twice a day until the animals showed autonomy and no infections detected. Manual bladder voiding was performed twice a day during the first week and once every day until sacrifice or spontaneous restoration of bladder control was achieved. Food pellets were provided on the cage floor during the first few days to allow easy access. Animals were also monitored for body temperature, correct scarring of the surgical incision, and recovery of general activities (grooming and nesting for example). Five days after surgery, the staples were removed, and the animals were regrouped to promote socialization and decrease anxiety and stress. Animals were monitored during the experiment for humane endpoints: wounds, autophagy behavior, or weight loss (>20% of their baseline weight).

### Locomotor analysis

The BMS test was used to evaluate locomotor behavior (25), 3 days post-injury and once a week thereafter for 37 days. The mice were placed in an open arena for 4 min, and their locomotor function was evaluated by two independent observers who were blinded to the experimental groups. Each animal was scored on a scale ranging from 0 to 9. Animals presenting a BMS score greater than 1 in the first BMS assessment (3 dpi) was excluded because of incomplete spinal cord compression.

### Bladder function

The bladders were manually voided and the animals were placed in the cage with water provided *ad libitum* overnight. Water weights in the cage bottles were measured before and after the experiment to assess water intake. Bladders were then voided into a beaker and the urine was weighed. The ratio between water intake and urine was calculated to assess bladder control in the different experimental groups. If the amount of urine was less than 0.1 g we considered that the animal regained total bladder control.

### Von Frey

The Von Frey test was used to determine tactile sensitivity by measuring how much force is required to elicit movement of the paw fingers, using the up-and-down method with Von Frey monofilaments, as previously described (26). The experimental setting consisted of placing the mice in an elevated mesh restrained inside a standard perforated box. Before the test started, each animal was habituated to the test conditions. A total of 9 monofilaments were used, ranging from 0.008 to 1.4 g. Both paws were stimulated with the central monofilament (0.16 g). If the animal moved the fingers of the paw, a weaker monofilament was used; otherwise, a stronger monofilament was applied. The test was performed until: 1) observed response to the 0.008 g monofilament, 2) no response to 1.4 g monofilaments, or 3) after a total of five measures around the threshold. 50% threshold was calculated using the formula:


$$50\%\text{ threshold}=\frac{10^{\left(x\_f+k\delta \right)}}{10000}$$

Where xf is the value of the final monofilament used (log units), K is the tabular value for the pattern of positive/negative responses, and δ is the mean difference between stimuli (0.267).

### Flow cytometry

Nine days post-injury, approximately 50 μL of blood was collected from the tail vein of the animals. Erythrocytes were depleted with ACK lysis solution. The cell pellet was then washed with FACS buffer (PBS, 10% BSA, 0.1% azide). 1x10^6^ cells were stained. The Fc portion was blocked using anti-mouse CD16/CD32 (Biolegend). Cell staining was performed by incubating a cocktail of antibodies for 30min at 4°C (**Table 2**). After washing, the cells were re-suspended in 200 μL FACS buffer. Precision counting beads (Biolegend) were added to the single-cell suspensions according to the manufacturer’s instructions to calculate the final cell concentrations. Cells were acquired using an LSRII Flow Cytometer (BD Pharminogen) and analyzed using Flow Jo software version 10.4. The gating strategy used can be found in the **Supplementary Data** (**Supplementary Figure S5**).

**Table 2:** Flow cytometry analysis summary of markers expressed on different cell populations.

Marker	Fluorochrome	Company	Target	Dilution
CD86	PerCpCy5.5	Biolegend	Myeloid cells	1/100
CD11b	PE	Biolegend	Myeloid cells	1/200
CD11c	BV 605	Biolegend	Mostly dendritic cells	1/100
NK 1.1	BV 510	Biolegend	Natural killer	1/100
CD19	FITC	Biolegend	B lymphocytes	1/200
CD3	APC	Biolegend	T lymphocytes	1/100
CD45	PeCy7	Biolegend	Leukocytes	1/200
Ly6C	BV711	Biolegend	Monocytes	1/100
Ly6G	BV650	Biolegend	Granulocytes	1/100
CD16/32	None	Biolegend	Fc Block	1/25

### Spinal cord collection, processing and immunohistochemistry

To understand the molecular and cellular effects of the different treatments on the spinal cord injury environment, an immunohistochemistry protocol to mark GFAP (astrocytes), Iba-1 (macrophages/microglia), PDGFR (fibrosis), and NeuN (mature neurons) was performed on mouse spinal cords. First, at 5 weeks post-injury mice were anesthetized and perfused with 20 mL of cold PBS and then with 4% PFA. A dorsal incision was made to remove the spinal cord with the vertebral column. The isolated spinal cords were then fixed with 4% PFA for 24h at 4°C. After, the tissue was placed on 30% saccharose solution until reaching saturation point, which was then cut into 1 cm fragments centered in the lesion site. Next, the spinal cords were embedded in optimal cutting temperature (OCT) solution and frozen in isopentane and liquid nitrogen. Using a Leica CM 1900 cryostat, the spinal cords were cut into transverse sections of 20 µm and mounted onto microscope slides (SuperFrost Plus) that were stored at -20°C for further use.

On the day of immunohistochemistry, slides with frozen sections were thawed at RT and cleaned with PBS to remove any remaining cryopreservation solution. This was followed by permeabilization with PBS-T 0.2% (v/v) for 10 min and a blocking solution of 5% (v/v) FCS in PBS-T 0.2% (v/v) for 30 min. An overnight incubation at 4°C was then performed with the following primary antibodies: rabbit anti-GFAP (1:200, DAKO), rabbit anti-Iba-1 (1:200, Wako), PDGFR (1:1000, Abcam), and rabbit anti-NeuN (1:200, D4G4O). The next day, after washing, the samples were incubated with Alexa Fluor 594 goat anti-rabbit (1:1000) (Abcam) secondary antibody for 3 h at RT. Cells were then counterstained with DAPI for 20 min before mounting the slides in Immu-Mount^®^ (Thermo Scientific) for subsequent image analysis. A negative control (primary antibodies omitted) was performed to discard any background as positive staining (**Supplementary Figure S6**).

Imaging was performed using an Olympus Widefield Inverted Microscope IX81. GFAP staining was evaluated by measuring the area of astrogliosis morphology, normalized to the total GFAP area. IBA-1 was evaluated by assessing the area of ramified macrophages/total microglia. Fibrosis was evaluated by assessing the area of PDGFR+ area normalized for total spinal cord area. The location of the spinocerebellar (SCT), rubrospinal (RST) and the corticospinal tracts (CST) were identified using the spinal cord atlas developed by Paxinos, Watson and Kayalioglu (27). The positive area for Thy1-GFP was calculated and divided for the total area of the tract in each spinal section. Positive Thy1-GFP and total areas were calculated using the plugin Neurite-J from the ImageJ (NIH) software as described above. NeuN staining was measured by counting the number of positive cells in laminae VIII and IX of both ventral horns.

### Proteomics analysis

The secretome was first concentrated (×100) using ultracentrifugation with falcons with 5 kDa cut-off filter (Vivaspin, GE Healthcare). A protein precipitation step using TCA to a final concentration of 20% was performed, and protein pellets were re-suspended in 35μL of Laemmli sample buffer. Protein extracts from each sample were separated by SDS-PAGE for approximately 16 min at 110 V (Short-GeLC Approach) (1) and stained with Coomassie Brilliant Blue G-250. Each lane was divided into three separate gel fractions for a destaining step using a solution of 50 mM ammonium bicarbonate with 30% acetonitrile, followed by overnight protein digestion with trypsin. Peptide extraction from the gel was performed using solutions containing different percentages of acetonitrile (30, 50, and 98%) with 1% of formic acid. For protein identification, each fraction was analyzed separately, and for protein quantification, fractions from each sample were combined, and a single analysis per sample was performed by LC-MS/MS.

Samples were analyzed on a NanoLC™ 425 System (Eksigent) coupled to a Triple TOF™ 6600 mass spectrometer (Sciex) and the ionization source (ESI DuoSpray™ Source). The chromatographic separation was performed on a Triart C18 Capillary Column 1/32” (12 nm, S-3μm, 150 x 0.3 mm, YMC) and using a Triart C18 Capillary Guard Column (0.5 × 5 mm, 3 μm, 12nm, YMC) at 50°C. The flow rate was set to 5 μL/min, and mobile phases A and B were 5% DMSO plus 0.1% formic acid in water and 5% DMSO plus 0.1% formic acid in acetonitrile, respectively. The LC program was performed as follows: 5 – 30% of B (0 - 50 min), 30 – 98% of B (50 - 52 min), 98% of B (52 - 54 min), 98 - 5% of B (54 - 56 min), and 5% of B (56 - 65 min). The ionization source was operated in the positive mode set to an ion spray voltage of 5500 V, 25 psi for nebulizer gas 1 (GS1), 10 psi for nebulizer gas 2 (GS2), 25 psi for the curtain gas (CUR), and source temperature (TEM) at 100°C. For data-dependent acquisition (DDA) experiments, the mass spectrometer was set to scanning full spectra (m/z 350-2250) for 250 ms, followed by up to 100 MS/MS scans (m/z 100 – 1500). Candidate ions with a charge state between +1 and +5 and counts above the minimum threshold of 10 counts per second were isolated for fragmentation, and one MS/MS spectrum was collected before adding those ions to the exclusion list for 15 s (mass spectrometer operated by Analyst^®^ TF 1.8.1, Sciex^®^). The rolling collision enErgy was used with a collision enErgy spread of 5. For SWATH experiments, the mass spectrometer was operated in a looped product ion mode and specifically tuned to a set of 42 overlapping windows, covering the precursor mass range of 350-1400 m/z. A 50 ms survey scan (350-2250 m/z) was acquired at the beginning of each cycle, and SWATH-MS/MS spectra were collected from 100-2250 m/z for 50 ms, resulting in a cycle time of 2.2 seconds.

Protein identification was performed using the ProteinPilot™ software (v5.0.2, Sciex) for each sample. The paragon method parameters were as follows: searched against the reviewed Mus musculus database from SwissProt, cysteine alkylation by acrylamide, digestion by trypsin, and gel-based ID. An independent False Discovery Rate (FDR) analysis using the target-decoy approach provided by Protein Pilot™, was performed to assess the quality of the identifications. SWATH data processing was performed using SWATH™ processing plug-in for PeakView™ (v2.0.01, Sciex^®^). Relative protein quantification was performed in all samples using information from the Ion-Library search. Quantification results were obtained for peptides with less than 1% of FDR for at least one of the samples by calculating the sum of up to five fragments/peptides. Relative peptide peak areas were normalized to the internal standard peak areas. Protein quantities were obtained by the sum of up to 15 peptides/proteins. Protein–protein interactions and network analysis was constructed using the online STRING database (https://string-db.org) version 11.5, depicting both functional and physical protein associations with a medium confidence level (0.4), and organized into clusters through k means clustering method. All identified proteins were then subjected to an over-representation analysis using the ConsensusPathDB. From a total of 368 proteins identified using LC-MS/MS, we focused the analysis on those that presented higher concentrations (fold changes of 2 or higher) between the two groups. These proteins were then grouped by function using the UniProt database and a heat map of their concentration was plotted with a cut off of 5 (ratios higher than 5 were color-expressed as 5). The mass spectrometry proteomics data have been deposited to the ProteomeXchange Consortium via the PRIDE (28) partner repository with the dataset identifier PXD048453.

### LEGENDplex

The concentration of relevant cytokines was evaluated in the secretomes of polarized macrophages using the LEGENDplex™ Mouse Macrophage/Microglia Panel kit according to manufacturer’s instructions. The secretome was first concentrated (×10) using ultracentrifugation with falcons with 5 kDa cut-off filter (Vivaspin, GE Healthcare). Then, reagents were prepared from the stocks provided, and standard serial dilutions were prepared to generate a standard curve. Assay buffer (25µL) was added to standard and sample wells in a 1:1 ratio. 25µL of mixed beads were added to each well, and the plate was incubated for 2 hours at RT with continuous agitation at 800 rpm. After a centrifugation of 250g for 5min, beads were washed with 1x wash buffer for 1min. 25µL of detection antibodies was added to each well, followed by 1 hour of incubation at RT with agitation at 800 rpm. 25µL of Streptavidin-phycoerythrin (SA-PE) was added directly to the previous solution, and the plate was incubated for 30 minutes at RT with agitation at 800 rpm. After a wash step with 150µL of 1x wash buffer, the samples were ready to read on the flow cytometer. For that, samples were vortexed, and 300 beads per analyte were acquired in a BD LSRII Flow Cytometer (BD, Pharminogen). The FCS files were analyzed using Biolegend’s LEGENDplex™ data analysis software site. Concentration values were subsequently divided by 10 to account for the concentration step, providing an accurate representation of the actual cytokine concentration present in the secretome.

### Statistical analysis

Statistical analyses were performed using GraphPad Prism software, version 8.0.1. The normality of the data was evaluated using the Shapiro-Wilk normality test. Gene expression, axonal regeneration *in vitro*, weight loss, bladder function, chronic pain, LEGENDplex, and flow cytometry data were analyzed using One-Way ANOVA followed by Tukey’s multiple comparison test. Data from the BMS score, astrogliosis, fibrosis, spinal tracts area, axonal arborization, and ramified microglia were assessed by two-way ANOVA followed by Tukey’s multiple comparison test. Live imaging data were assessed by unpaired, non-parametric t-test (Mann-Whitney test). Statistical significance was defined as p< 0.05 (95% confidence level). Data are presented as mean ± standard error (SEM).

## Results

### Monocytes isolation, differentiation and polarization

**Figure 1:**
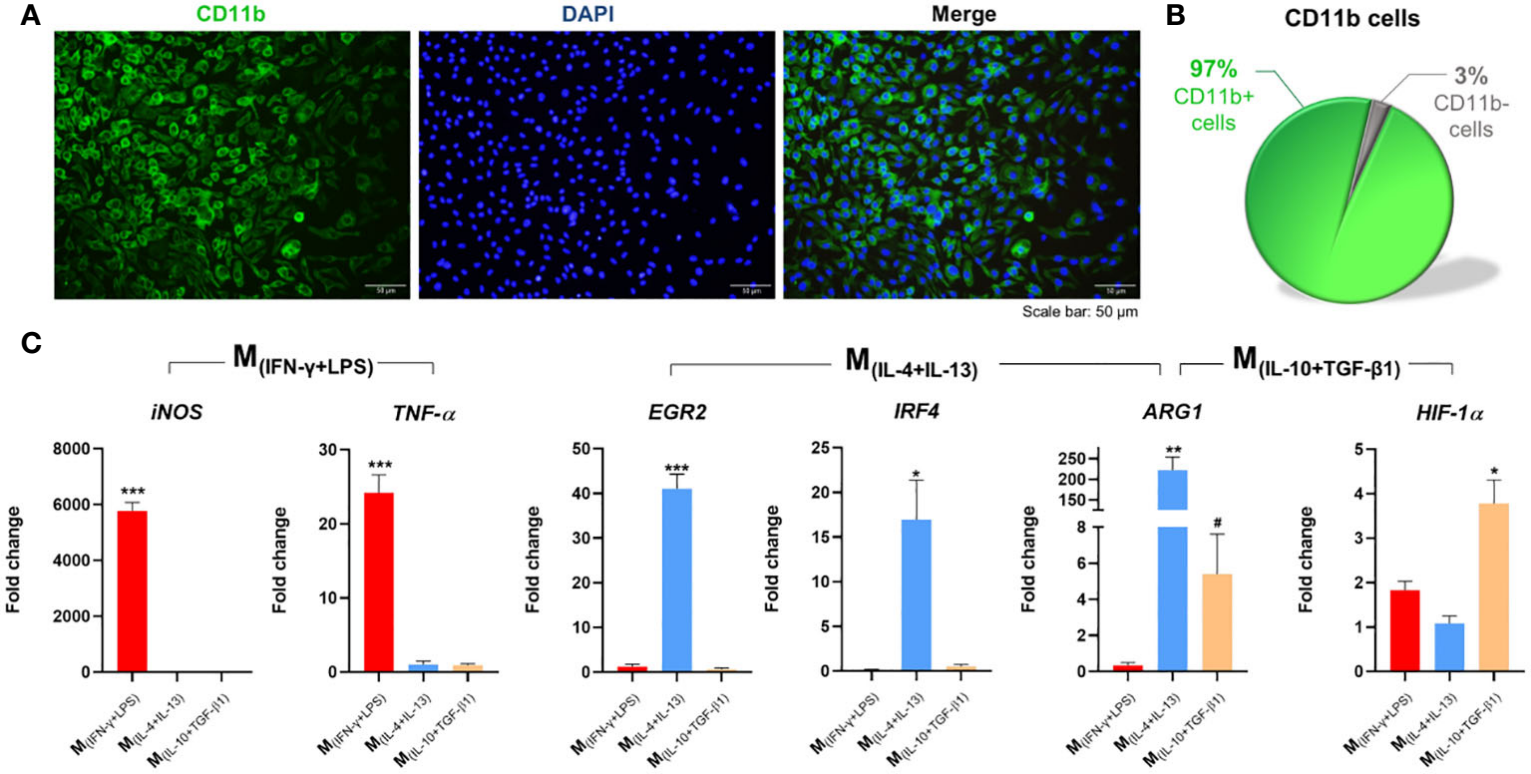
Isolation, differentiation, and polarization of macrophages. **(A)** Splenic monocytes cultured with macrophage colony-stimulating factor (M-CSF) for 7 days differentiated into macrophages; **(B)** with a culture purity of 97%. **(C)** Macrophages stimulated for 6 h with INF-γ and LPS significantly overexpressed *iNOS* (2, 7 df, p<0.0001) and *TNF-α* (2, 7 df, p<0.0001). Macrophages stimulated with IL-4 and IL-13 significantly overexpressed *EGR2* (2, 6 df, p<0.0001), *IRF4* (2, 6 df, p=0.0216), and Arg-1 (p=0.0020); and Macrophages stimulated with IL-10 and TGF-β1 significantly overexpressed ARG1 (p=0.0357), and *HIF-1α*.(2, 8 df, p=0.0032). Target genes were normalized to three reference genes: GADPH, HPRT and 18s. Fold-change levels were calculated by the 2-ΔΔct method related to non-stimulated macrophages. In immunocytochemistry photomicrographs macrophages were quantified using the anti-CD11b antibody (green) and nuclei were stained with DAPI (blue). One Way ANOVA followed with Tukey *post-hoc* test was used for statistical analysis. Arg-1 data were analyzed using the Mann Whitney test because normality was not achieved using the Shapiro-Wilk test. Data is presented as mean ± standard error (SEM). df= degrees of freedom, * or ^#^- p< 0.05; **- p< 0.01; ***- p< 0.001. Scale bar =50 µm. n=3. 2 independent experiments were performed.

To successfully culture spleen-derived macrophages (Sp-MФ), we isolated monocytes from the spleen and cultured them in the presence of macrophage colony-stimulating factor (M-CSF) to stimulate the survival, proliferation, and differentiation of monocytes into macrophages (**Figure 1A**). Using our protocol, we were able to obtain a highly enriched culture (97% purity) of Sp-MФ (**Figure 1B**). Without M-CSF, it was impossible to establish and maintain the cells (**Supplementary Figure S1A**), indicating that M-CSF is essential for the Sp-MФ culture.

To polarize macrophages into different phenotypes, we stimulated macrophages for 24h with 20 ng/mL of IFN-γ plus 100 ng/mL of LPS (classical activation) or with 20 ng/mL of IL-4 plus 20 ng/mL of IL-13 or 20 ng/mL of IL-10 plus 20 ng/mL of TGF-β1 (alternative activation). With immunocytochemistry it was possible to confirm that the classical activation leads to the polarization of 89% of the macrophages (**Supplementary Figure S1B**). Moreover, proteomics analysis of the secreted proteins of each macrophage population revealed that out of 487 proteins identified, 81 were exclusive secreted by M(_INF-γ+LPS_) macrophages, 35 by M(_IL-4+IL-13_), and 90 by M(_IL-10+TGF-β1_) macrophages (**Supplementary Figure S1C**). Using the Protein Analysis Through Evolutionary Relationships (PANTHER) tool, we further demonstrated distinctions in the protein classes among these populations of proteins (**Supplementary Figure S1D**). Metabolite interconversion enzymes were identified as a common protein class between the different macrophage populations, but as can be observed by the pie charts, the protein class or the percentage of proteins in different classes varied considerably among each cell phenotype (**Supplementary Figure S1D**). Additionally, the phenotypes of each macrophage population was also confirmed by gene expression analysis. qPCR revealed that Sp-MФ are easily polarized in vitro; namely, when macrophages were stimulated with IL-4+IL-13, they significantly overexpressed EGR2 and IRF4, and these genes were not overexpressed when macrophages were stimulated with IL-10+TGF-β1 (**Figure 1C**). The ARG1 gene was significantly overexpressed in the two populations of macrophages with alternative activation, however more overexpressed in the M_(IL-4+IL-13)_ phenotype than in the M_(IL-10+TGF-β1)_ macrophages. In contrast, the IL-10+TGF-β1 stimulation protocol led to a significant increase in HIF-1α expression, and these gene was not overexpressed with the IL-4+IL-13 stimuli. Using gene expression, we also confirm that macrophages under classic activation stimuli significantly overexpressed iNOS and TNF-α genes (**Figure 1C**). All these genes are known to be specifically overexpressed in these phenotypes (29). These results showed that we were able to obtain three different subsets of macrophages, one with classical activation (M_(INF-γ+LPS)_) and two with alternative activation (M_(IL-10+TGF-β1)_; M_(IL-4+IL-13)_).

### IL-10 and TGF-β1 activation promotes higher axonal growth

**Figure 2:**
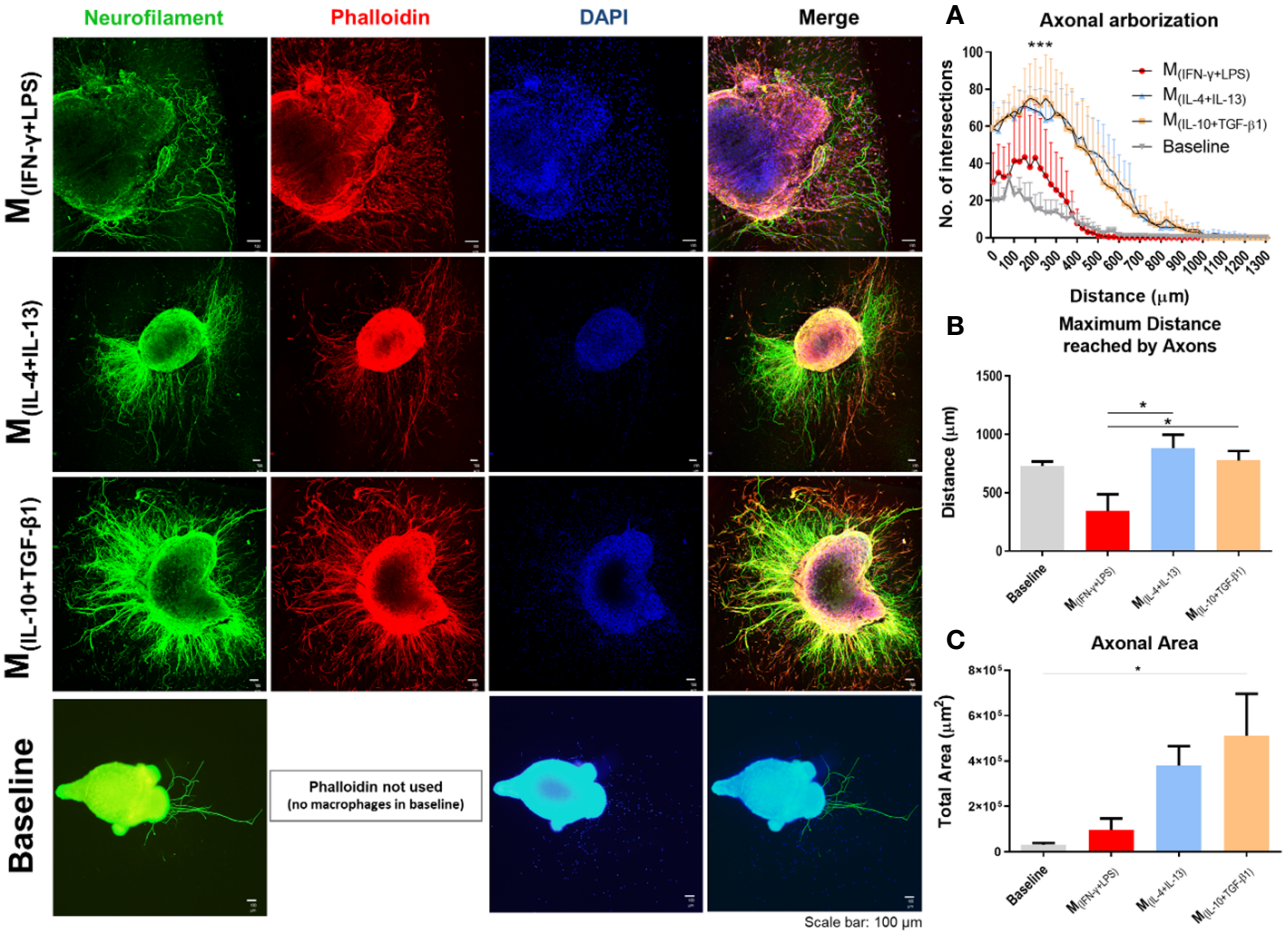
Classical (M_(INF-γ+LPS)_) or alternative (M_(IL-4+IL-13)_; M_(IL-10+TGF-β1)_) activated macrophages co-cultured with dorsal root ganglia (DRGs) in 3D collagen hydrogels. DRGs were stained with Neurofilament (green), Macrophages and DRGs stained with Phalloidin (red) and nuclei counterstained with DAPI (blue). **(A)** DRGs co-cultured with M_(IL-4+IL-13)_ and M_(IL-10+TGF-β1)_ macrophages had significantly higher axonal arborization (3, 12 df, p<0.0001) and **(B)** significantly longer axons (3, 14 df, p=0.0172) than M_(INF-γ+LPS)_ group and basal levels. **(C)** M_(IL-10+TGF-β1)_ condition also showed significant higher axonal area than basal levels (3, 14 df, p= 0.0292). Statistical analysis for axonal arborization employed two-way ANOVA followed by Tukey’s multiple comparisons test, while total area and distance were analyzed using one-way ANOVA followed by Tukey’s test. Data is presented as mean ± standard error (SEM). df= degrees of freedom, *- p< 0.05; ***- p< 0.001. Scale bar =100 µm; M_(IFN-γ+LPS)_ n= 3; M_(IL-4+IL-13)_ n=5; M_(IL-10+TGF-β1)_ n=5. 2 independent experiments were performed.

The effects of each macrophage subtype on axonal growth were then investigated. Spleen-derived macrophages polarized into M_(INF-γ+LPS)_, M_(IL-10+TGF-β1)_ or M_(IL-4+IL-13)_ were co-cultured with DRGs growing in three dimensions (**Figure 2**). DRGs cultured without macrophages were used as baseline. The results showed that DRGs co-cultured with M_(IL-10+TGF-β1)_ and M_(IL-4+IL-13)_ macrophages had significantly higher axonal arborization than those co-cultured with M_(INF-γ+LPS)_ or than basal levels (**Figure 2A**). DRGs co-cultured with M(_IL-10+TGF-β1_) and M(_IL-4+IL-13_) macrophages also presented significantly longer axons (**Figure 2B**) than those co-cultured with M_(INF-γ+LPS)_. Concerning the total axonal area, only M(_IL-10+TGF-β1_) condition showed significant differences from baseline (**Figure 2C**). We also performed a similar experiment with DRGs growing in two dimensions, which did not allow axonal growth in depth, but enabled direct contact between macrophages and DRGs (**Supplementary Figure S2**). Interestingly, under these conditions, only the DRGs co-cultured with M_(IL-10+TGF-β1)_ macrophages presented significantly higher axonal arborization (**Supplementary Figure S2A**) than those co-cultured with the other subtypes of macrophages, these DRGs also have significantly longest neurite (**Supplementary Figure S2B**), and higher axonal area (**Supplementary Figure S2C**) than M_(IL-4+IL-13)_ macrophages. It is important to point out that without the collagen matrix, axonal growth is significantly reduced, and not even the direct contact of the macrophages compensates for the absence of the 3D matrix.

**Figure 3:**
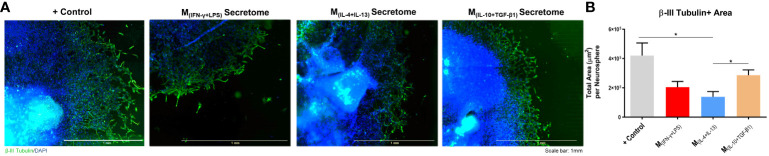
Axonal area of differentiated Neural Stem Cells obtained from human induced Pluripotent Stem Cells. **(A)** Axonal area was stained using anti-βIII tubulin antibody (green) and nuclei counterstained with DAPI (blue); **(B)** Statistical analysis demonstrated that the factors secreted by M_(IL-10+TGF-β1)_ macrophages are able to significantly preserve/regenerate the differentiated neurons (2, 18, p=0.0364) than the M_(IL-4+IL-13)_-secreted factors. One Way ANOVA followed by Tukey *post-hoc* test was used for statistical analysis. Data is presented as mean ± standard error (SEM). *- p< 0.05; M_(IFN-γ+LPS)_ n=6; M_(IL-4+IL-13)_ n=9; M_(IL-10+TGF-β1)_ n=6; +Ct n=9. Scale bar=1 mm. 2 independent experiments were performed.

**Figure 4:**
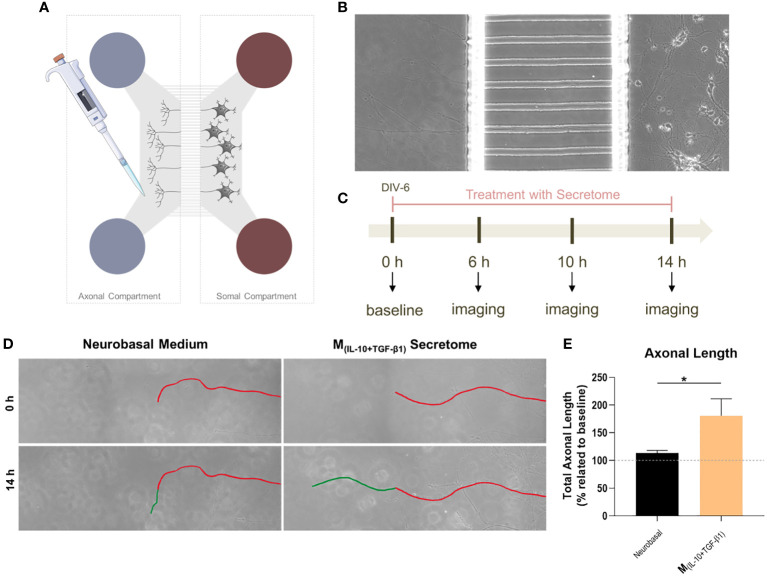
The effect of M_(IL-10+TGF-β1)_-derived secretome on the CNS neurons. **(A)** Schematic representation of the microfluidic chambers used. **(B)** Brightfield images of the axonal and somal compartments of the microfluidic chambers. **(C)** Schematic representation of the workflow used. **(D)** Brightfield images of axons growing under the effect of the soluble factors and extracellular vesicles secreted by M_(IL-10+TGF-β1)_ macrophages or Vehicle (Neurobasal Medium). In red is represented the length of the axon at baseline and in green after 14h of live imaging. **(E)** The secretome of M_(IL-10+TGF-β1)_ macrophages promotes significantly axonal regeneration (p= 0.0286). Statistical significance tested by unpaired, non-parametric t-test (Mann-Whitney test). Data is presented as mean ± standard error (SEM). *- p< 0.05; n=4, DIV= days *in vitro*. 2 independent experiments were performed.

The neuronal effects of the molecules and extracellular vesicles secreted by the different subtypes of splenic macrophages were also tested using human-derived neurospheres obtained from iPSCs. Neurospheres were allowed to differentiate into neurons for two days and then cultured with the secretome derived from each macrophage subtype (**Figure 3A**). It proved challenging to establish the baseline level of neuronal growth devoid of secreted factors as attempts to culture human neurospheres solely in basal medium were unsuccessful, leading to detachment from the culture plates and rendering meaningful analysis unfeasible. Nonetheless, we conducted a positive control using the regular culture medium to provide a comparative reference point. The total axonal area divided by the number of neurospheres was analyzed as described for the DRGs (see materials and methods section). As expected, the positive control group presented an overall neuronal area higher than all the groups, but notably it was only significantly different when compared with the M(_INF-γ+LPS_) and M(_IL-4+IL-13_) groups, but not with the M(_IL-10+TGF-β1_)-derived secretome (**Figure 3B**). Results also demonstrated that the M_(IL-10+TGF-β1)_ secretome significantly promoted more axon preservation/regeneration than M_(IL-4+IL-13)_ secretome (**Figure 3B**). Both subtypes are pro-regenerative; however, our in vitro results showed that the M_(IL-10+TGF-β1)_-derived secretome has higher regenerative capabilities. For this reason, we then tested only the secretome derived from this sub-population in CNS-derived neurons. Primary cortical neurons were plated in the soma compartment of microfluidic chambers (**Figures 4A, B**), and neuronal growth was live imaged (**Supplementary Video 1**) in the axonal compartment for 14h (**Figures 4C, D**). The results demonstrated that M_(IL-10+TGF-β1)_-derived secretome promoted significant axonal regeneration compared with the control medium (**Figure 4E**).

### M_(IL-10+TGF-β1)_ derived secretome promotes functional recovery *in vivo*

**Figure 5:**
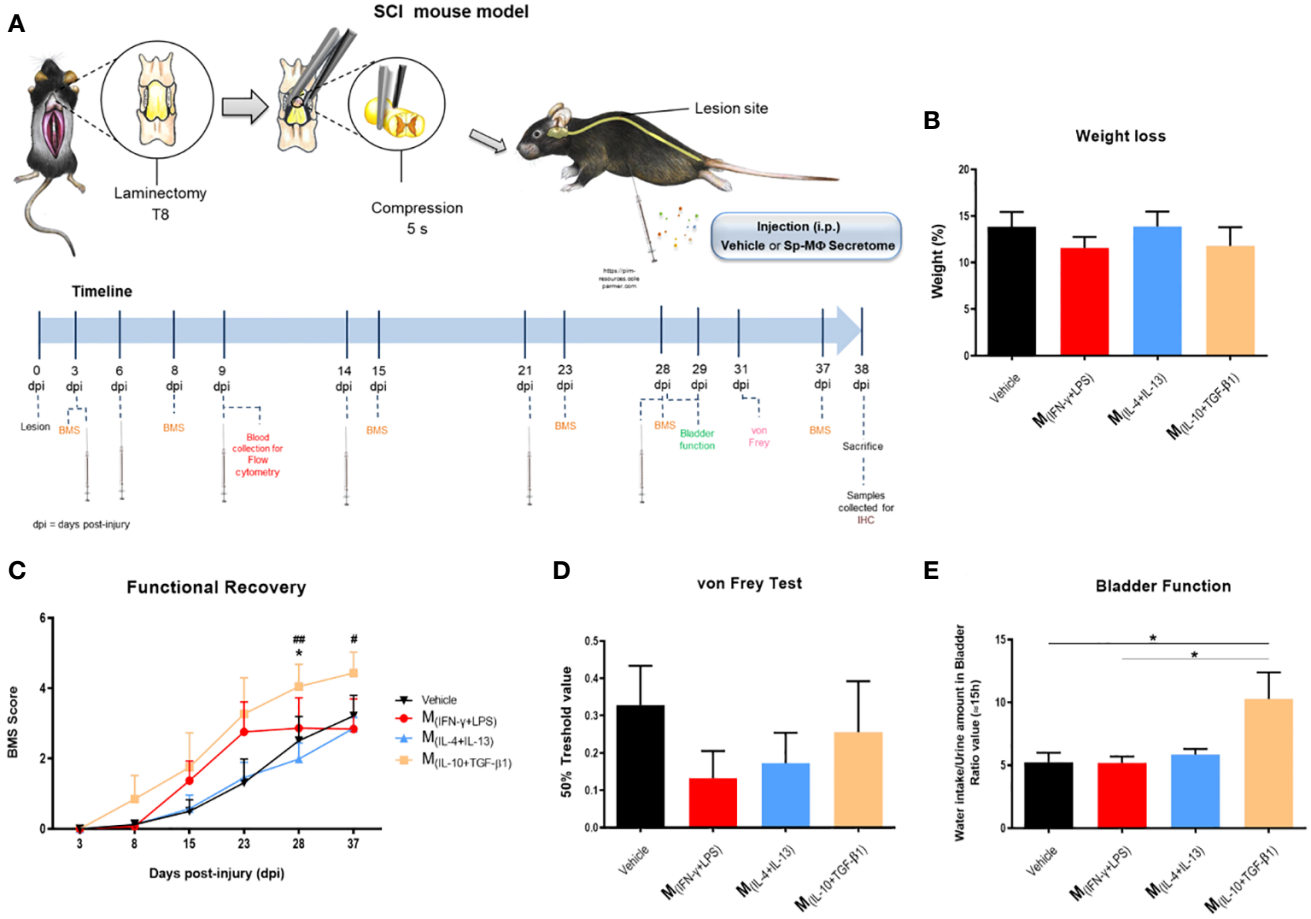
Pre-clinical evaluation of macrophages derived secretome using a SCI compression model. **(A)** Schematic layout of the *in vivo* testing. **(B)** The treatment had no effect on weight of the animals 38 dpi (3, 25 df, p=0.6013). **(C)** Animals treated with M_(IL-10+TGF-β1)_-derived secretome presented significantly better functional scores than the other treatment groups, namely than the Vehicle (3, 25 df, p=0.0465) and M_(IL-4+IL-13)_ group (3, 25 df, p= 0.0047) at 28 days and the M_(IL-4+IL-13)_ at 37 days (3, 25 df, p= 0.0359). **(D)** No significantly differences were observed on the hypersensitivity of the animals 38 dpi, however, M_(INF-γ+LPS)_-treated mice presented a tendency to be more hypersensitive (3, 25 df, p=0.5097). **(E)** Animals treated with M_(IL-10+TGF-β1)_-derived secretome presented significant recovery of the bladder function when assessed 38 dpi (3, 22 df, p= 0.0137). Two-way repeated measure ANOVA was used to analyze statistical differences on the BMS data and One-Way ANOVA was used to analyze statistical differences on the other functional tests followed by the multiple comparison test Tukey. Data is presented as mean ± standard error (SEM). * or #- p< 0.05; ##- p< 0.01; df= degrees of freedom, Vehicle n=8; M_(INF-γ+LPS)_ n=7; M_(IL-4+IL-13)_ n=8; M_(IL-10+TGF-β1)_ n=6. 1 independent experiment was performed.

In vitro experiments demonstrated that the soluble factors and extracellular vesicles secreted by macrophages may have therapeutic potential for neural repair. Therefore, we tested whether intraperitoneal injections (500 µL) of macrophage-derived secretome could be used as a therapy for spinal cord injury. Forty-two mice were subjected to compression SCI and 3, 6, 9, and 14 days post-injury (and then once a week up to 28 dpi) were treated with secretome derived from different macrophage subtypes (**Figure 5A**). During the experimental protocol, all animals lost weight without significant differences between groups (**Figure 5B**). To evaluate motor function, we performed the BMS test, in which a higher score indicates higher motor recovery. We found that mice treated with M_(IL-10+TGF-β1)_ secretome had significantly higher BMS scores than those treated with the vehicle or M_(IL-4+IL-13)_ (**Figure 5C**). Only animals treated with this pro-regenerative cocktail (M_(IL-10+TGF-β1)_ secretome) were able to perform weight-supported plantar stepping, while the other treatment regimens only led to extensive ankle movement recovery without weight support. Interestingly, in the first 2/3 weeks post-injury, mice treated with the pro-inflammatory cocktail (M_(INF-γ+LPS)_ secretome) presented a functional recovery very close to those treated with the M_(IL-10+TGF-β1)_ secretome, indicating that this pro-inflammatory cocktail may be beneficial in the early phase. However, continuing with M_(INF-γ+LPS)_ secretome treatment, the functional recovery stabilized, and the therapeutic effect disappeared (**Figure 5C**), indicating that the non-resolving nature of chronic exposure to this pro-inflammatory cocktail is detrimental. Four weeks post-injury, we performed the von Frey filament test to assess the mechanical sensitivity function of the animals. We did not detect any statistical differences; however, mice treated with the pro-inflammatory cocktail (M_(INF-γ+LPS)_ secretome) had lower values, indicating that this treatment may lead to hypersensitivity. In contrast, the vehicle and M_(IL-10+TGF-β1)_ secretome groups showed higher values in the von Frey filament test (**Figure 5D**), indicating less hypersensitivity. We also analyzed mouse autonomic function, namely bladder function, using the ration between water intake and amount of urine in the bladder. Bladder recovery is an important priority for people living with SCI (30). Our results showed that mice treated with the M_(IL-10+TGF-β1)_ secretome had a significant recovery of bladder control compared to those treated with vehicle and M_(INF-γ+LPS)_ secretome (**Figure 5E**). This preclinical trial demonstrated that the therapeutic effect of the molecules and extracellular vesicles secreted by the different subtypes of macrophages varies depending on the phenotype, even when using two pro-regenerative phenotypes.

### M_(IL-10+TGF-β1)_ derived secretome modulates pathophysiological events leading to neuronal survival *in vivo*

**Figure 6:**
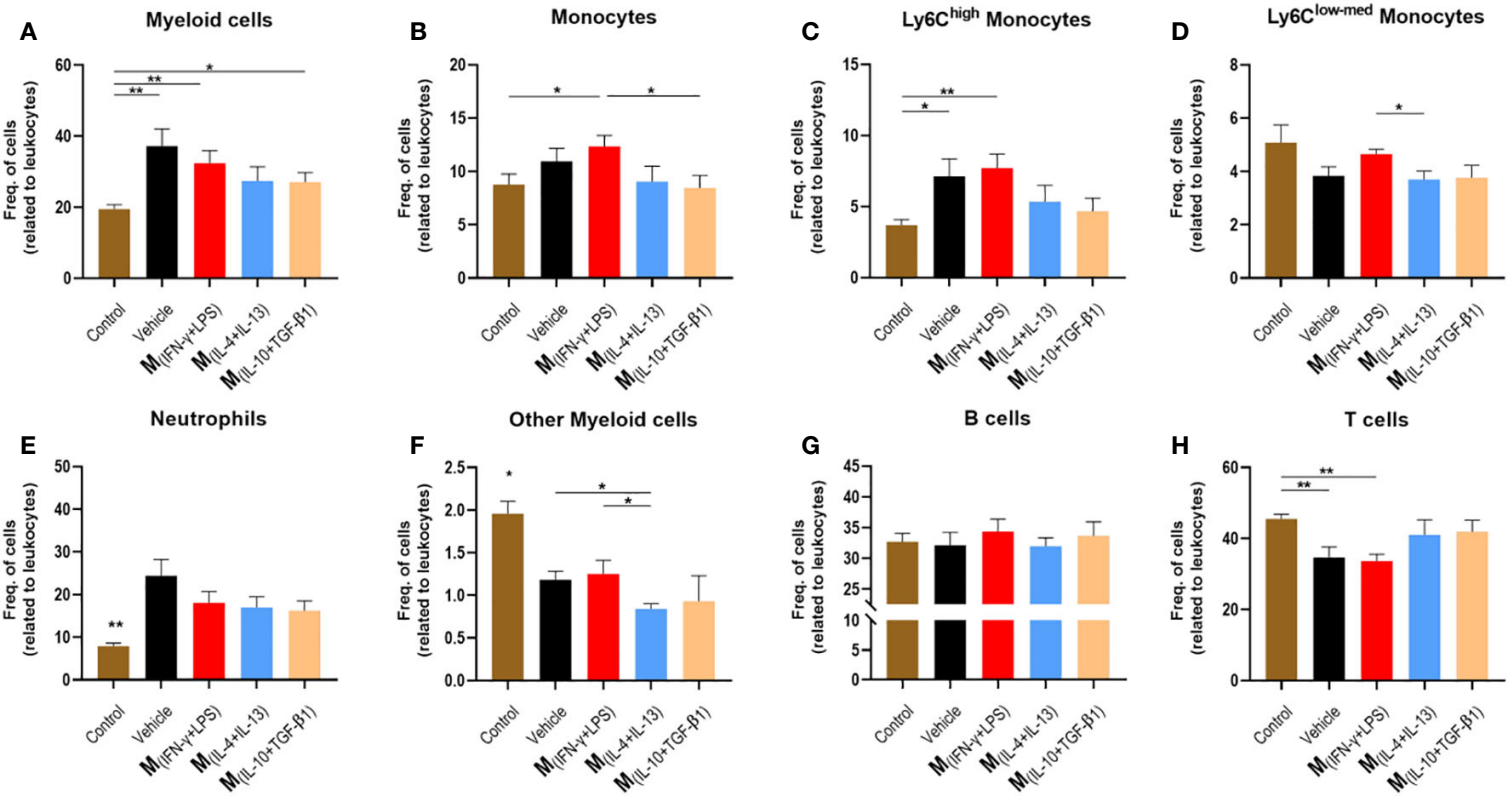
Leukocytes in circulation 9 days post injury. Blood was collected from the tail vein and process for analysis using flow cytometry. **(A)** Animals treated with vehicle, M_(INF-γ+LPS)_ and M_(IL-10+TGF-β1)_-derived secretome had significantly higher myeloid cells than control mice (4, 17 df, p=0.0240). **(B)** Animals treated with M_(INF-γ+LPS)_-derived secretome had significantly higher frequency of monocytes than M_(IL-10+TGF-β1)_ secretome group and control (4, 17 df, p=0.0457). **(C)** Animals treated with vehicle or M_(INF-γ+LPS)_-derived secretome had a significantly higher frequency of Ly6C^high^ monocytes (4, 17 df, p=0.0282) than control mice. **(D)** M_(INF-γ+LPS)_ group also had significantly more Ly6C^med+low^ monocytes than M_(IL-4+IL-13)_-treated mice (4, 17 df, p=0.0457). **(E)** Animals without a SCI had significantly lower frequency of Neutrophils (4, 17 df, p=0.0055). **(F)** M_(IL-4+IL-13)_-treated mice had significantly less other myeloid cells than vehicle and M_(INF-γ+LPS)_-treated animals (4, 17 df, p=0.0358). **(G)** No differences were observed for B Cells (4, 17 df, p=0.8721) and **(H)** M_(INF-γ+LPS)_ and vehicle-treated mice had significantly lower frequency of T cells (4, 17 df, p=0.0011) than control mice. One-Way ANOVA was used to analyze statistical differences followed by the multiple comparison test Tukey. Data is presented as mean ± standard error (SEM). *- p< 0.05; **- p< 0.01; df= degrees of freedom, Control n= 5; Vehicle n=5; M_(INF-γ+LPS)_ n=4; M_(IL-4+IL-13)_ n=5; M_(IL-10+TGF-β1)_ n=4. 1 independent experiment was performed.

To understand the effect of the secretome on the immune response, we collected blood from all groups nine days post-injury and used healthy mice as controls. Flow cytometry was used to verify the inflammatory profile of leukocytes in circulation, which could infiltrate the injured spinal cord. Analysis revealed that mice treated with vehicle, M_(INF-γ+LPS)_ and M_(IL-10+TGF-β1)_ secretome had a significantly higher frequency of myeloid cells in circulation (**Figure 6A**). Mice treated with the M_(INF-γ+LPS)_ secretome had a significantly higher frequency of monocytes than the M_(IL-10+TGF-β1)_ secretome (**Figure 6B**). Mice treated with vehicle or M_(INF-γ+LPS)_ secretome had a significantly higher frequency of Ly6C^high^ monocytes (**Figure 6C**). It is noteworthy that the Ly6C^high^ monocytes are prone to become pro-inflammatory macrophages (31). M(_INF-g+LPS_) also presented significantly more Ly6Clow-med monocytes in circulation (**Figure 6D**). All animals with SCI had significantly more circulating neutrophils (**Figure 6E**). Concerning the rest of myeloid cells, M(_INF-g+LPS_) also presented significant increase (**Figure 6F**). No differences were observed between the groups for B cells (**Figure 6G**). M_(INF-γ+LPS)_ and vehicle-treated mice had a significantly lower frequency of T cells (**Figure 6H**). Of note, the number of animals used in the flow cytometry analysis varies from that used in functional recovery data because we opted to spare some animals during this sub-acute phase due to their weakened state. To conduct flow cytometry of circulating leukocytes blood collection was necessary, we decide to prioritize the well-being of the animals avoiding unnecessary risks of losing mice.

**Figure 7:**
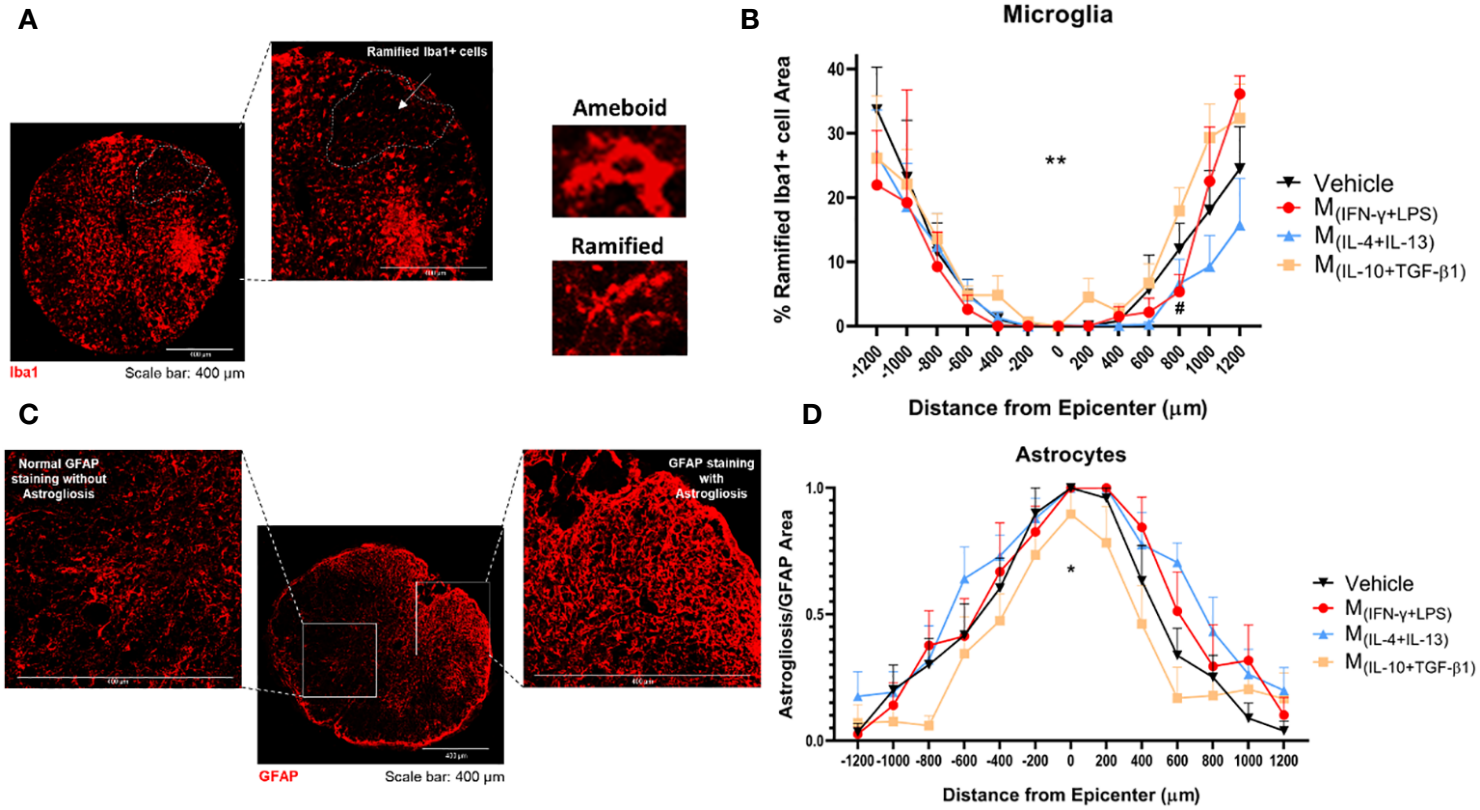
Histological analysis of the spinal cord 38 dpi. **(A)** Representative image of microglia from M_(IL-10+TGF-β1)_-treated group, cells were stained using the antibody anti-IBA1 (red) and the area of the ramified microglia was analyzed. **(B)** Rostral-caudal analysis demonstrated that the animals treated with M_(IL-10+TGF-β1)_-derived secretome presented overall significantly more ramified microglia than M_(IL-4+IL-13)_ group (3, 239 df, p=0.0058) and presented more ramified microglia than the M_(INF-γ+LPS)_ group at 800 µm caudal to the injury (3, 239 df, p=0.0469). **(C)** Representative image of astrocytes from vehicle-treated group, cells were stained with anti-GFAP antibody (red) and astrogliosis were analyzed by quantification of the area of clustered GFAP overstaining (areas impossible to distinguish individual astrocytes). **(D)** Rostral-caudal analysis demonstrated that mice treated with M_(IL-10+TGF-β1)_-derived secretome had significantly less astrogliosis than the animals treated with M_(IL-4+IL-13)_ or M_(INF-γ+LPS)_ secretome (3, 231 df p<0.0001). Differences in both microglia and astrocytes analysis were detected using two-way ANOVA followed by Tukey’s multiple comparisons test. A total of 284 spinal cord slices were observed to analyze astrogliosis and 301 slices to microglia. Data is presented as mean ± standard error (SEM). * or #- p< 0.05; **- p< 0.01; Vehicle n=7; M_(INF-γ+LPS)_ n=6; M_(IL-4+IL-13)_ n=8; M_(IL-10+TGF-β1)_ n=6. 1 independent experiment was performed.

Thirty-eight days post-injury, the animals were sacrificed and the spinal cords were collected for histological analysis. IBA-1antibody was used to study the morphology of microglia and distinguish between ramified and amoeboid microglia (**Figure 7A**). Rostral-caudal analysis of the spinal cord showed that mice treated with the pro-regenerative cocktail M_(IL-10+TGF-β1)_ had a significantly higher percentage of ramified microglia than mice treated with the pro-inflammatory cocktail M_(INF-γ+LPS)_, or the M_(IL-4+IL-13)_-secreted factors (**Figure 7B**).

The GFAP antibody was used to analyze astrogliosis. Areas of clustered GFAP overstaining were considered astrogliosis (**Figure 7C**). The analysis revealed that mice treated with the pro-regenerative cocktail, M_(IL-10+TGF-β1)_ secretome, had significantly lower astrogliosis (**Figure 7D**), which indicate that these animals presented diminished scar.

Neurons from the ventral horns, namely from lamina VIII and IX, were counted using an anti-NeuN antibody (**Supplementary Figure S3A**). Mice treated with M_(IL-10+TGF-β1)_ secretome have significantly more neurons than animals treated with the pro-inflammatory-derived secretome (**Supplementary Figure S3B**). Finally, concerning fibrosis, the rostral-caudal analysis did not detect significant differences in the PDGFR+ area (**Supplementary Figure S3C**) between the treated groups when all areas of the spinal cord were analyzed (**Supplementary Figure S3D**); however, caudally to the injury epicenter, mice treated with the M_(IL-10+TGF-β1)_ secretome have significantly less fibrosis than the other treatments (**Supplementary Figure S3E**).

### M_(IL-10+TGF-β1)_ derived secretome preserved ascending and descending spinal tracts after SCI

**Figure 8:**
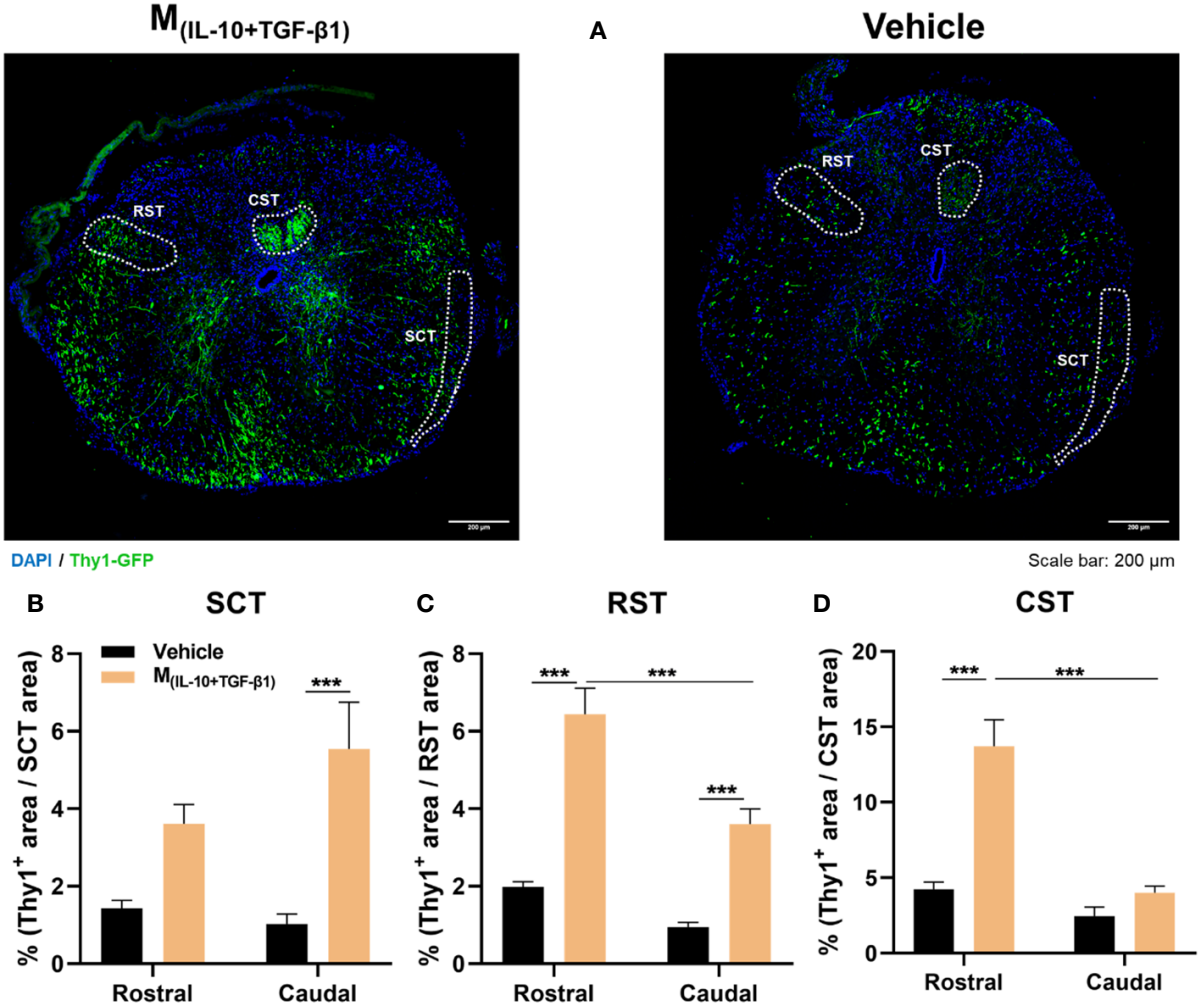
Histological analysis of ascending and descending spinal tracts 38 dpi. **(A)** Representative images of Thy1-GFP animals from M_(IL-10+TGF-β1)_ and Vehicle-treated group. The positive area for Thy1-GFP (green) was calculated and divided for the total area of the tract in each spinal section. The analysis encompassed a range spanning 600 µm to 2000 µm in both rostral and caudal directions from the epicenter. **(B)** The secretome derived from M_(IL-10+TGF-β1)_ macrophages significantly promoted higher neuronal preservation of spinocerebellar tract (SCT) in the caudal region (3, 65 df, p=0.0007) when compared with vehicle treatment. **(C)** Animals treated with M_(IL-10+TGF-β1)_ secretome also revealed a higher preservation of the rubrospinal tract (RST) both in the rostral (3, 64 df, p<0.0001) and in the caudal region (3, 64 df, p=0.0010). Moreover, the treatment effect was significantly higher in the rostral region than in the caudal (3, 64 df,<0.0001). **(D)** Likewise, the M_(IL-10+TGF-β1)_ secretome significantly preserved the corticospinal tract (CST) descending axons, namely rostrally from the epicenter (3, 74 df, p<0.0001), and this preservation was significantly higher in the rostral than in the caudal region (3, 74 df, p<0.0001). Two-way ANOVA followed by Tukey’s multiple comparisons test was used to analyze statistical differences. A total of 163 spinal cord slices were analyzed. Data is presented as mean ± standard error (SEM). ***- p< 0.001. Vehicle n=3; M_(IL-10+TGF-β1)_ n=3. 1 independent experiment was performed.

Considering the functional and histological outcomes obtained from our pre-clinical trial, we executed a subsequent in vivo protocol with a focused objective: to assess the therapeutic efficacy of M(IL-10+TGF-β1) secretome specifically in the preservation of spinal tracts critical for locomotion. These tracts include the corticospinal tract (CST), rubrospinal tract (RST), and spinocerebellar tract (SCT). Within this cohort of animals, we employed mice harboring the Thy1-GFP transgene, and the percentage of positive area for Thy1-GFP in each distinct spinal tract was calculated (**Figure 8A**). The analysis encompassed a range spanning 600 µm to 2000 µm in both rostral and caudal directions from the epicenter. The vicinity of the epicenter was excluded from the analysis due to challenges in pinpointing the exact location of the spinal tracts. Results demonstrated that the administration of M_(IL-10+TGF-β1)_ secretome significantly contributes to the preservation of the spinocerebellar tract caudally to the epicenter (**Figure 8B**), both the rostral and caudal portions of the rubrospinal tract (**Figure 8C**), and the preservation of the corticospinal tract (**Figure 8D**) in the rostral region. Significant differences were also observed between the rostral and caudal regions, however, only in the motor tracts (RST and CST). Specifically, the rostral regions exhibited a markedly higher extent of neuronal preservation in comparison to the caudal regions (**Figures 8C, D**).

### M_(IL-10+TGF-β1)_ secretome present molecules involve with anti-inflammatory, phagocytosis and tissue repair/remodeling processes

**Figure 9:**
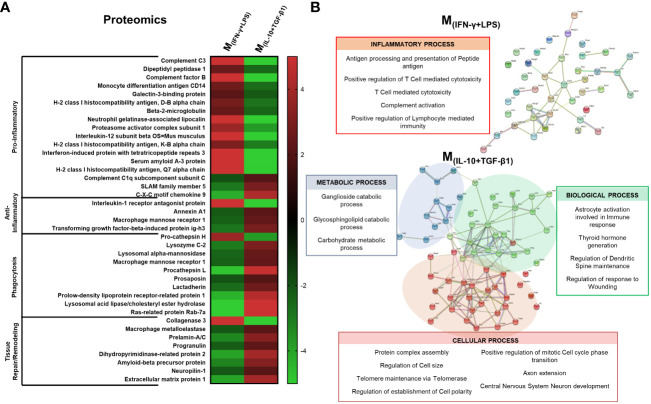
Proteomic analysis by LC-MS/MS focused on the proteins that presented higher concentration in the secretome. **(A)** 14 out of 17 pro-inflammatory proteins were overconcentrated in M_(INF-γ+LPS)_-derived secretome; 3 out of 4 anti-inflammatory proteins were overconcentrated in M_(IL-10+TGF-β1)_ secretome; M_(IL-10+TGF-β1)_ secretome was enriched in proteins involved on phagocytosis (9 out of 10) and in proteins involved in tissue repair/remodeling (7 out of 8). **(B)** Cluster analysis using the STRING database revealed that the secretome of M_(INF-γ+LPS)_ macrophages only presented proteins related to the inflammatory process (antigen processing and presentation of peptide antigen, T cell mediated cytotoxicity and complement activation); M_(IL-10+TGF-β1)_ macrophages secreted proteins were classified into three main clusters: Cluster 1 - proteins related with metabolic process; Cluster 2 - proteins that participate in biological processes, such as, astrocyte activation, involved in immune response, regulation of dendritic spine maintenance and regulation of response to wounding; and Cluster 3 - proteins that play a role in cellular processes such as axon extension and central nervous system neuron development. Proteins were identified using the KEGG Orthology database. 1 independent experiment was performed.

In order to understand which proteins secreted by the different phenotypes of macrophages could be important for the differences observed both in vitro and in vivo, we identified and quantified the proteins produced by the macrophages using both the bead-based immunoassay LEGENDplex and liquid chromatography with mass spectrometry (LC-MS/MS). LC-MS/MS allows a broader and non-target analysis; however, it may not detect small and low-concentrated proteins, such as cytokines and chemokines. For this reason, we complemented LC-MS/MS analysis with the immunoassay LEGENDplex. The results demonstrated that pro-inflammatory cytokines such as TNF-α, G-CSF and IL12p40 were present almost only in the secretome of M_(INF-γ+LPS)_ and were significantly different from the other groups (**Supplementary Figure S4**). These results were expected because these cytokines are characteristic of proinflammatory macrophages. Additionally, the cytokine/hormone G-CSF was also significantly elevated in the M_(INF-γ+LPS)_ secretome (**Supplementary Figure S4**). In turn, TGF-β1, a cytokine with anti-inflammatory properties, was present in higher quantities in the M_(IL-10+TGF-β1)_ and M_(IL-4+IL-13)_ subsets; however, only in the pro-regenerative phenotype M_(IL-10+TGF-β1)_ that this cytokine reached significant differences (**Supplementary Figure S4**). The macrophage subsets that presented interesting results both in vivo and in the LegendPlex assay were the M_(IL-10+TGF-β1)_ and M_(INF-γ+LPS)_ phenotypes, and therefore, a detailed proteomics analysis was only performed in the secretome derived from these two populations. From a total of 452 proteins identified, we focused the analysis on those that presented higher concentrations (fold changes of 2 or higher) between the two groups. These proteins were grouped by function using the UniProt database, and the results revealed that 14 out of 17 pro-inflammatory proteins were overconcentrated in the M_(INF-γ+LPS)_ secretome, and 3 out of 4 anti-inflammatory proteins were overconcentrated in the M_(IL-10+TGF-β1)_ secretome (**Figure 9A**). Moreover, the M_(IL-10+TGF-β1)_ secretome was also enriched in proteins involved in phagocytosis (9 out of 10) and in proteins involved in tissue repair/remodeling (7 out of 8) (**Figure 9A**). These results were expected because the M_(INF-γ+LPS)_ and M_(IL-10+TGF-β1)_ subsets are classified as pro-inflammatory and anti-inflammatory/repairing, respectively. Finally, protein–protein interaction network analysis was constructed using the online STRING database depicting both functional and physical protein associations and the results revealed that the secretome of M_(INF-γ+LPS)_ contains proteins from just one cluster, which can be considered a cluster related to the inflammatory process, since these proteins are involved in antigen processing and presentation of peptide antigen, T cell-mediated cytotoxicity, and complement activation (**Figure 9B**). In contrast, the M_(IL-10+TGF-β1)_ secretome contained proteins from three different clusters, a cluster of proteins more related to metabolic processes (Cluster 1), with proteins that participate in Ganglioside and Glycosphingolipid catabolic processes (**Figure 9B**). Two other clusters were identified, with proteins that participate in relevant biological and cellular processes, such as astrocyte activation involved in immune response, regulation of dendritic spine maintenance, and regulation of response to wounding (Cluster 2), and proteins that play a role in axon extension and central nervous system neuron development (Cluster 3), some of which may be responsible for the improvements observed in vivo (**Figure 9B**).

## Discussion

After injury, the immune system is fundamental for promoting adequate tissue repair and regeneration. However, it is well known that the immune response after SCI is dysfunctional and is an important contributor to the secondary damage observed after primary injury. Several therapeutic approaches have been designed to shut down the immune response after SCI; however, more important than shutting it down is to transform a dysfunctional response into a regenerative one. After SCI, splenic and bone marrow-derived monocytes infiltrate the lesion site and differentiate into macrophages (4). There is abundant literature exploring bone marrow-derived monocytes in an SCI context (10, 32–34), however, less is known about splenic monocytes. The spleen is not just important for erythrocyte recycling and immune response to pathogens. After injury, immune cells in the spleen become rapidly activated and mobilize to sites of damaged tissue. This activation and mobilization was first observed after myocardial ischemia and also demonstrated after SCI (5, 24). Splenic monocytes infiltrated the spinal cord in the acute phase of the injury, peaking at 7 days, whereas bone marrow-derived monocytes only infiltrated the cord 1 week after injury (4). Although the spleen has been characterized as the major source of pro-inflammatory monocytes after SCI (4), in ischemic brain injury models, splenic monocytes have been demonstrated to be key effector cells that modulate meningeal and parenchymal immune responses and limit ischemic injury, leading to improved functional outcomes (35). This indicates a complex interplay between the recruited splenic monocytes and the tissue microenvironment that finally determines the macrophage phenotype.

For these reasons, in this work, we aimed to study and further characterize splenic-derived macrophages in an SCI context, as this cell population may play a key role in tissue repair.

In this study, we used a protocol that led to a highly pure (97%) culture of primary splenic macrophages without the need to use cell sorting or magnetic beads separation kits. It is difficult to compare our purity with other protocols in the literature because the vast majority of studies do not disclaim this value (36–38) or use macrophage cell lines instead of primary cells (39). We demonstrated that splenic monocytes are similar to monocytes from other origins in terms of plasticity and are easily polarized into pro-inflammatory or pro-regenerative phenotypes. Moreover, we demonstrated that different splenic macrophage phenotypes have distinct effects on axonal growth and neuroprotection. Namely, classical activation (pro-inflammatory) has a detrimental impact, whereas alternative activation promotes axonal regeneration and neuroprotection. To the best of our knowledge, these biological effects were first described in our work for spleen-derived macrophages; however, these effects were also previously demonstrated in bone marrow-derived macrophages (10, 40). It is important to point out that the vast majority of the research in the literature only studied one type of alternative activation of macrophages (using IL-4); herein, we showed that activation with TGF-β1 and IL-10 has significantly superior biological effects than activation with IL-4 and IL-13, not only *in vitro* but also in an *in vivo* SCI model.

As previously mentioned, the microenvironment at the SCI site favors predominant and sustained macrophage polarization into a pro-inflammatory phenotype, which is detrimental to tissue repair (15). Some authors have investigated the therapeutic effect of transplanting alternatively activated macrophages into the damaged spinal cord to balance the ratio between pro- and anti-inflammatory macrophages at the injury site (12, 32). However, clinical trials have failed to demonstrate a significant therapeutic effect. Clinical results did not support the treatment of acute SCI with autologous incubated macrophage therapy (14). The reason behind this disappointing result may be that transplanted macrophages fail to retain their pro-regenerative phenotype when transplanted into the injured spinal cord (10). Kroner and colleagues demonstrated that intracellular accumulation of iron by macrophages induces a rapid switch from a pro-regenerative to a pro-inflammatory phenotype in the spinal cord tissue (15). Therefore, in this study, we decided to inject the soluble factors and extracellular vesicles produced by macrophages (secretome) instead of transplanting the cells. Herein, we explored whether systemic injections of secretomes derived from different macrophage phenotypes have a therapeutic effect after SCI. We tested the complete secretome rather than separating the soluble and vesicular fractions, because our previous evidence demonstrated that for SCI repair, the secretome as a whole is advantageous over the individual fractions (41). The local immune response after SCI is known to be dysfunctional; however, SCI also leads to the systemic dysregulation of the immune response. For instance, it was demonstrated that SCI could promote pro-inflammatory responses that damage peripheral organs (42, 43). Moreover, our group previously demonstrated that the infiltration of neutrophils into the injured spinal cord is affected by neural communication between the spinal cord and the spleen (24). The combined factors of local environment and systemic dysregulation of the immune response led us to choose the systemic administration of secretome instead of local administration or local transplantation of macrophages. In this way, we not only avoided losing the phenotype of the transplanted cells due to local environmental cues, but we are also able to modulate/prime immune cells even before they infiltrate the spinal cord. Notably, in our experimental animal model, the blood-spinal cord barrier (BSCB) is disrupted due to the mechanical compression, allowing the systemic-injected molecules to reach the spinal cord tissue. However, it is crucial to acknowledge that, even with this scenario, the majority of systemically delivered secretome is directed towards peripheral organs such as the liver, lungs, and spleen (44–46). Moreover, in some clinical scenarios, the BSCB may remain intact, in these situations intrathecal administration may be necessary.

In this study, we observed that the M_(IL-10+TGF-β1)_-derived secretome is the most effective treatment in promoting functional recovery after compressive SCI. Additionally, factors and extracellular vesicles secreted by M_(IL-10+TGF-β1)_ also supported the recovery of bladder function. Regain of bladder control is an important functional priority for persons living with SCI (30, 47). Interestingly, up to 3 weeks post-injury, treatment with the pro-inflammatory secretome, M_(INF-γ+LPS)_, had a similar therapeutic effect to the M_(IL-10+TGF-β1)_ secretome; however, the continued injection of molecules derived from the pro-inflammatory phenotype was shown to be detrimental in the long term. In line with this observation, previous research performed by Freria and colleagues demonstrated that preconditioning microglia with LPS injection before ischemic SCI elicits reactive spinal cord microglia and confers neuroprotection, leading to functional recovery (48). Indeed, a pro-inflammatory response seems to be necessary, at least in the acute phase or before injury; however, our results show that if this pro-inflammatory stimulus continues over time, the therapeutic effect ceases and becomes disadvantageous. We also observed that animals treated with the pro-inflammatory secretome tend to have more neuropathic pain. This data is in accordance with the current literature demonstrating that inflammation in the spinal cord leads to mechanical allodynia (49, 50). Microglia activation in the spinal cord is critical for developing pain hypersensitivity through the production of pro-inflammatory cytokines, chemokines and extracellular proteases (51). Activated microglia directly interacts with nociceptors and interneurons by modulating cell surface receptors and ion channels (52).

The identification and quantification of the molecules on the secretome were studied using flow cytometry, through the Legendplex immunoassay kit, and proteomic analysis using LC-MS/MS. Proteomics data were further examined using the STRING database, a web-based open resource that analyzes all known and predicted associations between proteins, including physical and functional interactions (53). Cluster analysis of the M_(INF-γ+LPS)_-derived secretome revealed that only one class of proteins was functionally enriched. Namely, proteins associated with a pro-inflammatory response, such as molecules related to positive regulation of T cells cytotoxicity and lymphocytes, mediate immunity, as well as complement activation molecules and proteins involved in antigen processing and presentation of peptide antigen. The immunoassay also revealed that the cytokines TNF-α, IL-12p40, and G-CSF were enriched in the M_(INF-γ+LPS)_ secretome. On the other hand, analysis of the M_(IL-10+TGF-β1)_-derived secretome showed that these macrophages secrete a wide variety of proteins structured in three main functional clusters: 1) proteins involved in phagocytosis; 2) proteins involved in tissue remodeling/response to wounding; and 3) proteins with anti-inflammatory properties. Moreover, STRING analysis identified clusters of proteins on the M_(IL-10+TGF-β1)_ secretome involved in axon extension, dendritic spine maintenance, establishment of cell polarity, and regulation of astrocytic activation. Looking for individual proteins enriched in the M_(IL-10+TGF-β1)_ secretome, it is possible to find some proteins with a known effect after SCI. For instance, it was demonstrated that Anexinn 1a administration decreased caspase-3 and IL-1β expression, reduced tissue damage, and protected axons of long descending pathways *in vivo* (54). In this context, the presence of Anexinn 1a within the secretome likely contributed to the preservation of long descending and ascending spinal tract. Our findings underscore the capacity of the M_(IL-10+TGF-β1)_ secretome to significantly support the structural integrity of crucial neuronal tracts, including the ascending spinocerebellar tract (SCT) and the descending rubrospinal (RST) and corticospinal tracts (CST). Notably, these tracts assume pivotal roles in locomotion. For instance, the significance of SCT neurons in orchestrating the genesis and perpetuation of locomotor behavior in both neonatal and adult mice has been previously described (55). SCT neurons exhibit inherent rhythmogenic attributes and intricate circuit connectivity with spinal interneurons within the locomotor central pattern generator (55). Moreover, the indispensability of this neuronal pathway for motor function restoration in human individuals afflicted with spinal cord injuries has been well documented (56–58).

Likewise, the RST plays a multifaceted role in various components of dexterous motor functions. Disruptions within the RST give rise to deficits in intricate motor tasks such as reaching and grasping, as well as stepping movements (59). Evidently, the structural soundness of the RST is indispensable for limb coordination during activities encompassing food retrieval and ambulation. Equally pivotal, the contribution of CST neurons to voluntary movement has been extensively elucidated (60, 61), as has the paramount importance of this spinal tract in effecting motor recovery in SCI patients (62, 63). It is important to note that a higher degree of neuronal preservation was observed within regions that continue to receive afferent neuronal input. Consequently, the rostral portions of the descending tract demonstrate a superior level of neuronal preservation compared to their caudal counterparts due to the enduring reception of supraspinal information. In contrast, the ascending tract exhibits an inverse relationship, wherein higher preservation is evident in caudal regions due to the persistence of afferent input.

Progranulin is another protein enriched in the M_(IL-10+TGF-β1)_ secretome, which may play a key role in repairing the injured spinal cord. Progranulin deficiency has been demonstrated to promote neuroinflammation and apoptosis and exacerbate damage (64). Moreover, progranulin protects lysosomal function and enhances the autophagic flux of microglia, allowing these cells to acquire an anti-inflammatory phenotype (65) and modulate the expression of GFAP, thereby decreasing the pro-inflammatory activation of astrocytes (66, 67). Indeed, previous studies have demonstrated that microglia respond rapidly to pathological stimuli, influencing then the fate of astrocytes (68, 69). Additional, using single-cell RNA sequencing, Brennan and colleagues revealed that microglia play a pivotal role in controlling stereotypical astrocyte-specific functions triggered by SCI, including upregulation of inflammatory genes, lipid processing, cell adhesion, and proliferation (70). Pro-inflammatory microglia release IL-1β, TNF-α, and complement component 1 subcomponent q (C1q), inducing the formation of inflammatory reactive astrocytes, commonly referred to as A1. Conversely, anti-inflammatory microglia promote the induction of pro-regenerative astrocytes, known as A2, thereby mitigating inflammation and exerting neuroprotective effects (68). Our histological analysis revealed that systemic injections of M_(IL-10+TGF-β1)_ secretome resulted in fewer amoeboid microglia and reduced astrogliosis in the spinal cord tissue 5 weeks post-injury. The factors present in the secretome likely influenced the microglial phenotype, leading to decreased astrogliosis.

TGF-β1 is elevated in the M_(IL-10+TGF-β1)_ secretome; however, its role after SCI is more controversial. Some studies have stated that TGF-β1 might have a detrimental role after SCI (71, 72), while others have shown that it may have a therapeutic role (73, 74). One study described TGF-β1 as an inducer and promoter of fibroblasts distribution and fibrotic scar formation (72). However, in this study we specifically analyzed the fibrotic scar and observed a significantly reduction of fibrosis on M_(IL-10+TGF-β1)_-treated animals; therefore, the systemic administration or the presence of other molecules on the secretome seems to inhibit this effect of TGF-β1 on fibrosis. One possible explanation for this finding is that it may be an indirect effect mediated by the modified microglia, similar to the mechanism observed in astrogliosis. It was demonstrated that microglia activated with anti-inflammatory factors can attenuate neuroinflammation-induced scarring by rescuing the expression of Arf and Rho GAP adapter protein 3 (75). Additionally, transplantation of neonatal microglia and single-cell RNA sequencing studies have highlighted the crucial role of microglia in scar-free healing (76). It is also important to point out that PDGFR+ cells may play a multifaceted role after spinal cord injury, with conflicting findings reported in the literature. As a major pericyte marker, PDGFRβ has been associated with the proliferation of scar-forming cells (77). Studies suggest that inhibiting the proliferation of PDGFRβ+ pericytes reduces fibrotic scar formation by fibroblasts, thereby promoting axon regeneration and functional recovery following SCI (78). On the contrary, evidence also indicates a positive role for PDGFRβ+ pericytes in sealing the lesion core after SCI, aiding in injury containment and protecting neural tissue (77, 79). However, it was demonstrated that PDGFR+ cells that contribute to normal tissue healing and regeneration return to their physiological niche, and that their prolonged presence in the tissue resulted in tissue fibrosis and aberrant healing (80). Our analysis was performed 38 days after injury, which may indicate that these cells are contributing to tissue fibrosis instead of tissue healing.

Finally, Dihydropyrimidinase-related protein 2, also known as Collapsin Response Mediator Protein-2 (CRMP2), is recognized for its affinity for tubulin heterodimers and functions in regulating the microtubule network, playing an important role in neuronal polarity establishment and axonal guidance (81). Several authors have identified CRMP2 as a crucial molecule for axonal regeneration (82, 83). The presence of this protein in the M_(IL-10+TGF-β1)_ secretome may be crucial for explaining the regeneration observed when using DRGs. *In vivo* CRMP2 was also identified as a contributor to the maintenance of spinal-cord regenerative ability (84), playing a key role in promoting axonal regeneration and leading to functional motor improvements (85). Recently, the function of CRMP2 was also described in human cells. The GADD45G/p38 MAPK/CDC25B signaling pathway promotes dephosphorylation of phosphorylated CRMP2 which in turn facilitates microtubule polymerization and leads to neurite outgrowth in human neurons (86).

Identifying the mechanism of action of our therapeutic approach is challenging; most likely, several proteins and extracellular vesicles have a distinct therapeutic action over time. Nonetheless, future experiments will focus on blocking some of the most promising candidates to understand whether the beneficial effects of the M_(IL-10+TGF-β1)_ secretome have one or several origins. In the first week after SCI, most of the monocytes circulating in the blood will be derived from the spleen reservoir (4), so in a putative clinical situation there is no need to obtain monocytes from the spleen of the person with SCI, a sample of blood will work. However, in future experiments, we will also have to test whether the M_(IL-10+TGF-β1)_ secretome obtained from monocytes isolated from blood has the same therapeutic action as those obtained directly from the spleen. Finally, in this study, we started the treatment 3 days after injury, which means that in a clinical scenario patients need to receive injections of the allogeneic-derived secretome. For autologous treatment, we will need to assess whether the M_(IL-10+TGF-β1)_ secretome maintains its therapeutic effect when administered at least 10 days post-injury.

## Conclusions

In this study, we demonstrated that different splenic macrophage phenotypes secrete factors and extracellular vesicles with distinct therapeutic effects. We conclude that systemic injection of the M_(IL-10+TGF-β1)_ secretome is the most effective treatment in promoting functional motor recovery after compressive SCI. Additionally, the M_(IL-10+TGF-β1)_ secretome supported the recovery of bladder function. Proteomic analysis showed that these macrophages secrete a wide variety of proteins involved in axon extension, dendritic spine maintenance, establishment of cell polarity, and regulation of astrocytic activation. The results presented herein are promising, and additional research is needed to optimize and characterize this therapy so that it can be translated to clinical use.

## Data availability statement

The original contributions presented in the study are included in the article/**Supplementary Material**. Further inquiries can be directed to the corresponding author. The mass spectrometry proteomics data have been deposited to the ProteomeXchange Consortium via the PRIDE partner repository with the dataset identifier PXD048453.

## Ethics statement

The animal study was approved by ethical Subcommittee in Life and Health Sciences (SECVS; ID:018/2019, University of Minho). The study was conducted in accordance with the local legislation and institutional requirements.

## Author contributions

JL-G: Formal analysis, Investigation, Writing – original draft. DS: Formal analysis, Investigation, Writing – review & editing. JA: Formal analysis, Investigation, Writing – review & editing. AM: Writing – review & editing. AP: Formal analysis, Investigation, Writing – review & editing. VM: Formal analysis, Investigation, Writing – review & editing. MD: Formal analysis, Investigation, Writing – review & editing. EG: Investigation, Writing – review & editing. RL: Investigation, Writing – review & editing. LF: Formal analysis, Investigation, Writing – review & editing. FF-A: Formal analysis, Investigation, Writing – review & editing. IP: Investigation, Writing – review & editing. NdS: Investigation, Writing – review & editing. JRC: Investigation, Writing – review & editing. AF: Investigation, Writing – review & editing. SS: Investigation, Writing – review & editing. LR: Investigation, Writing – review & editing. JC: Formal analysis, Investigation, Writing – review & editing. TP: Investigation, Writing – review & editing. SM: Investigation, Writing – review & editing. BM: Formal analysis, Writing – review & editing. AS: Funding acquisition, Writing – review & editing. RA: Formal analysis, Investigation, Writing – review & editing. NS: Conceptualization, Funding acquisition, Project administration, Resources, Supervision, Writing – original draft, Writing – review & editing.

## Supplementary material

Supplementary Figure 1 Splenic macrophages characterization. **(A)** MCS-F is essential for the survival and proliferation and differentiation of splenic monocytes into macrophages. **(B)** After 24h of polarization with the pro-inflammatory molecules LPS and INF-γ, 89% of the macrophages expressed iNOS. **(C)** A total of 487 proteins identified, 81 were exclusive to the secretome of M(_INF-γ+LPS_) macrophages, 35 to M(_IL-4+IL-13_), and 90 to M(_IL-10+TGF-β1_). **(D)** Using the PANTHER tool was possible to identified metabolite interconversion enzymes (dark blue) as a common protein class between the different macrophage populations, but as can be observed by the pie charts, the protein class and the percentage of proteins in different classes varied considerably among each cell phenotype. Anti-CD11b antibody was used to identify macrophages (green), anti-iNOS antibody was used to confirm the polarization (red) and nuclei was counterstained with DAPI (blue). Scale bar =50 µm. 2 independent experiments were performed for the *in vitro* data and 1 independent experiment for proteomics analysis.

Supplementary Figure 2 Classical (M_(INF-γ+LPS)_) or alternative (M_(IL-4+IL-13)_; M_(IL-10+TGF-β1)_) activated macrophages co-cultured with dorsal root ganglia (DRGs) in 2D. DRGs stained with Neurofilament (green), Macrophages and DRGs stained with Phalloidin (red) and nuclei counterstained with DAPI (blue). **(A)** DRGs co-cultured with M_(IL-10+TGF-β1)_ macrophages had significantly higher axonal arborization (3, 17 df, p<0.0001) and **(B)** significantly longer axons (p=0.0247) than the than M_(IL-4+IL-13)_. **(C)** M_(IL-10+TGF-β1)_ also had significant more axonal area (0.0240) than the M_(IL-4+IL-13)_. Two way ANOVA followed by Tukey *post-hoc* test was used for axonal arborization analysis and Kruskal-Wallis test followed by Dunn’s multiple comparisons test was used for longer distance and axonal area analysis. Data is presented as mean ± standard error (SEM). *- p< 0.05; ***- p< 0.001. Scale bar =200 µm; M_(INF-γ+LPS)_ n=5; M_(IL-4+IL-13)_ n=4; M_(IL-10+TGF-β1)_ n=5. 2 independent experiments were performed.

Supplementary Figure 3 Histological analysis of the spinal cord. **(A)** Representative image of gray matter neurons from M_(IL-10+TGF-β1)_-treated group, cell bodies were measured by counting the number of positive NeuN cells (red) in laminae VIII and IX of both ventral horns. **(B)** Rostral-caudal analysis demonstrated that the secretome derived from M_(IL-10+TGF-β1)_ cells significantly promoted neuronal survival at the ventral horns (3, 253 df, p= 0.0438) when compared with M_(IFN-γ+LPS)_. **(C)** Representative image of fibrotic scar from vehicle-treated group, anti-PDGFRβ antibody (red) was used to analyze fibrosis in the spinal cord. **(D)** Although there are not significant differences in PDGFRβ+ total area between treated groups, **(E)** rostral caudal analysis show that mice treated with M_(IL-10+TGF-β1)_-derived secretome had significantly less fibrosis caudally to the injury area (3, 49 df, p= 0.0370). ANOVA followed by the Tukey *post-hoc* test was used to analyze statistical differences. A total of 312 spinal cord slices were observed to analyze neuronal survival and 134 slices (53 for the caudal calculation) for fibrosis. Data is presented as mean ± standard error (SEM). *- p< 0.05. Vehicle n=7; M_(INF-γ+LPS)_ n=6; M_(IL-4+IL-13)_ n=8; M_(IL-10+TGF-β1)_ n=6. 1 independent experiment was performed.

Supplementary Figure 4 LEGENDplex immunoassay. The pro-inflammatory cytokines TNF-α, G-CSF and IL12p40 were significantly concentrated on the secretome of M_(INF-γ+LPS)_. The cytokine/hormone G-CSF was also significantly concentrated on the M_(INF-γ+LPS)_-derived secretome. TGF-β1, a cytokine with anti-inflammatory properties was significantly concentrated in the M_(IL-10+TGF-β1)_-derived secretome. Data was analyzed using the two-way ANOVA (2, 130 df, p<0.0001) followed by the Tukey’s multiple comparisons test. Data is presented as mean ± standard error (SEM). *- p< 0.05; ***- p< 0.001, M_(IFN-γ+LPS)_ n=4; M_(IL-4+IL-13)_ n=6; M_(IL-10+TGF-β1)_ n=6. Concentration values plotted in the graph were divided by 10 to account for the concentration step performed before the analysis. The values for CXCL-1, IL-12p70, and IL-10 were below the limit of detection and were consequently excluded from the analysis. 1 independent experiment was performed.

Supplementary Figure 5 Gating strategy used for flow cytometry analysis of mice blood cells. Doublets were excluded by FSC-A *vs* FSC-H scatter. Blood total cells were gated by SSC-A *vs* FSC-A scatter. Leukocytes were gated by CD45+ cells and on this population lymphocytes and myeloid cells were distinguished by CD11b expression. In lymphocytes population, CD3+CD19- cells were defined as T cells and CD3-CD19+ cells were defined as B cells. In myeloid cell population, Ly6G *vs* Ly6C allowed the selection of neutrophils (Ly6G+Ly6C+) and monocytes (Ly6G-Ly6C+). The selection of eosinophils *vs* monocytes Ly6C intermediate *vs* monocytes Ly6C high was made based on Ly6C *vs* SSC-A gating.

Supplementary Figure 6 Negative control fluorescence images of the Alexa Fluor 594 goat anti-rabbit antibody (red) both at the injury site and 800μm from the injury site. DAPI (blue) was used as structural marker.

## References

[1] Silva NA Sousa N Reis RL Salgado AJ. From basics to clinical: a comprehensive review on spinal cord injury. Prog Neurobiol (2014) 114:25–57. doi: 10.1016/j.pneurobio.2013.11.002 24269804

[2] Silva NA Sousa RA Pires AO Sousa N Salgado AJ Reis RL. Interactions between Schwann and olfactory ensheathing cells with a starch/polycaprolactone scaffold aimed at spinal cord injury repair. J BioMed Mater Res A (2012) 100:470–6. doi: 10.1002/jbm.a.33289 22125000

[3] Milich LM Ryan CB Lee JK. The origin, fate, and contribution of macrophages to spinal cord injury pathology. Acta neuropathologica (2019) 137:785–97. doi: 10.1007/s00401-019-01992-3 PMC6510275 30929040

[4] Blomster LV Brennan FH Lao HW Harle DW Harvey AR Ruitenberg MJ. Mobilisation of the splenic monocyte reservoir and peripheral CX(3)CR1 deficiency adversely affects recovery from spinal cord injury. Exp Neurol (2013) 247:226–40. doi: 10.1016/j.expneurol.2013.05.002 23664962

[5] Swirski FK Nahrendorf M Etzrodt M Wildgruber M Cortez-Retamozo V Panizzi P. Identification of splenic reservoir monocytes and their deployment to inflammatory sites. Science (2009) 325:612–6. doi: 10.1126/science.1175202 PMC2803111 19644120

[6] Gensel JC Zhang B. Macrophage activation and its role in repair and pathology after spinal cord injury. Brain Res (2015) 1619:1–11. doi: 10.1016/j.brainres.2014.12.045 25578260

[7] Galli SJ Borregaard N Wynn TA. Phenotypic and functional plasticity of cells of innate immunity: macrophages, mast cells and neutrophils. Nat Immunol (2011) 12:1035–44. doi: 10.1038/ni.2109 PMC3412172 22012443

[8] Mosser DM Edwards JP. Exploring the full spectrum of macrophage activation. Nat Rev Immunol (2008) 8:958–69. doi: 10.1038/nri2448 PMC2724991 19029990

[9] Shechter R Schwartz M. CNS sterile injury: just another wound healing? Trends Mol Med (2013) 19:135–43. doi: 10.1016/j.molmed.2012.11.007 23279948

[10] Kigerl KA Gensel JC Ankeny DP Alexander JK Donnelly DJ Popovich PG. Identification of two distinct macrophage subsets with divergent effects causing either neurotoxicity or regeneration in the injured mouse spinal cord. J Neurosci (2009) 29:13435–44. doi: 10.1523/JNEUROSCI.3257-09.2009 PMC2788152 19864556

[11] Monteiro S Salgado AJ Silva NA. Immunomodulation as a neuroprotective strategy after spinal cord injury. Neural Regener Res (2018) 13:423–4. doi: 10.4103/1673-5374.228722 PMC5900502 29623924

[12] Schwartz M Lazarov-Spiegler O Rapalino O Agranov I Velan G Hadani M. Potential repair of rat spinal cord injuries using stimulated homologous macrophages. Neurosurgery (1999) 44:1041–5. doi: 10.1097/00006123-199905000-00057 10232537

[13] Schwartz M Yoles E. Macrophages and dendritic cells treatment of spinal cord injury: from the bench to the clinic. Acta Neurochir Suppl (2005) 93:147–50. doi: 10.1007/3-211-27577-0_25 15986745

[14] Lammertse DP Jones LA Charlifue SB Kirshblum SC Apple DF Ragnarsson KT. Autologous incubated macrophage therapy in acute, complete spinal cord injury: results of the phase 2 randomized controlled multicenter trial. Spinal Cord (2012) 50:661–71. doi: 10.1038/sc.2012.39 22525310

[15] Kroner A Greenhalgh AD Zarruk JG Passos dos Santos R Gaestel M David S. TNF and increased intracellular iron alter macrophage polarization to a detrimental M1 phenotype in the injured spinal cord. Neuron (2014) 83:1098–116. doi: 10.1016/j.neuron.2014.07.027 25132469

[16] Pinho AG Cibrão JR Silva NA Monteiro S Salgado AJ. Cell secretome: basic insights and therapeutic opportunities for CNS disorders. Pharm (Basel) (2020) 13(2):31. doi: 10.3390/ph13020031 PMC7169381 32093352

[17] Livak KJ Schmittgen TD. Analysis of relative gene expression data using real-time quantitative PCR and the 2(-Delta Delta C(T)) Method. Methods (2001) 25:402–8. doi: 10.1006/meth.2001.1262 11846609

[18] Gomes ED Mendes SS Leite-Almeida H Gimble JM Tam RY Shoichet MS. Combination of a peptide-modified gellan gum hydrogel with cell therapy in a lumbar spinal cord injury animal model. Biomaterials (2016) 105:38–51. doi: 10.1016/j.biomaterials.2016.07.019 27505621

[19] Gomes ED Mendes SS Assuncao-Silva RC Teixeira FG Pires AO Anjo SI. Co-transplantation of adipose tissue-derived stromal cells and olfactory ensheathing cells for spinal cord injury repair. Stem Cells (2018) 36:696–708. doi: 10.1002/stem.2785 29352743

[20] Pinto MJ Pedro JR Costa RO Almeida RD. Visualizing K48 ubiquitination during presynaptic formation by ubiquitination-induced fluorescence complementation (UiFC). Front Mol Neurosci (2016) 9:43. doi: 10.3389/fnmol.2016.00043 27375430 PMC4901079

[21] Pinto MJ Alves PL Martins L Pedro JR Ryu HR Jeon NL. The proteasome controls presynaptic differentiation through modulation of an on-site pool of polyubiquitinated conjugates. J Cell Biol (2016) 212:789–801. doi: 10.1083/jcb.201509039 27022091 PMC4810304

[22] Rocha LA Gomes ED Afonso JL Granja S Baltazar F Silva NA. *In vitro* evaluation of ASCs and HUVECs co-cultures in 3D biodegradable hydrogels on neurite outgrowth and vascular organization. Front Cell Dev Biol (2020) 8:489. doi: 10.3389/fcell.2020.00489 32612997 PMC7308435

[23] Percie du Sert N Hurst V Ahluwalia A Alam S Avey MT Baker M. The ARRIVE guidelines 2.0: Updated guidelines for reporting animal research. BMC veterinary Res (2020) 16:242. doi: 10.1186/s12917-020-02451-y PMC7359286 32660541

[24] Monteiro S Pinho AG Macieira M Serre-Miranda C Cibrao JR Lima R. Splenic sympathetic signaling contributes to acute neutrophil infiltration of the injured spinal cord. J Neuroinflamm (2020) 17:282. doi: 10.1186/s12974-020-01945-8 PMC7513542 32967684

[25] Basso DM Fisher LC Anderson AJ Jakeman LB McTigue DM Popovich PG. Basso Mouse Scale for locomotion detects differences in recovery after spinal cord injury in five common mouse strains. J Neurotrauma (2006) 23:635–59. doi: 10.1089/neu.2006.23.635 16689667

[26] de Sousa N Pinho AG Monteiro S Liberato V Santos DJ Campos J. Acute Baclofen administration promotes functional recovery after spinal cord injury. Spine J (2022) 23(3):379–91. doi: 10.1016/j.spinee.2022.09.007 36155240

[27] Watson C Paxinos G Kayalioglu G Heise C. Chapter 16 - atlas of the mouse spinal cord. In: Watson C Paxinos G Kayalioglu G, editors. The Spinal Cord. Academic Press, San Diego (2009). p. 308–79.

[28] Perez-Riverol Y Bai J Bandla C García-Seisdedos D Hewapathirana S Kamatchinathan S. The PRIDE database resources in 2022: a hub for mass spectrometry-based proteomics evidences. Nucleic Acids Res (2022) 50:D543–d552. doi: 10.1093/nar/gkab1038 34723319 PMC8728295

[29] Lu J Cao Q Zheng D Sun Y Wang C Yu X. Discrete functions of M2a and M2c macrophage subsets determine their relative efficacy in treating chronic kidney disease. Kidney Int (2013) 84:745–55. doi: 10.1038/ki.2013.135 23636175

[30] Lo C Tran Y Anderson K Craig A Middleton J. Functional priorities in persons with spinal cord injury: using discrete choice experiments to determine preferences. J Neurotrauma (2016) 33:1958–68. doi: 10.1089/neu.2016.4423 27080545

[31] Kalinski AL Yoon C Huffman LD Duncker PC Kohen R Passino R. Analysis of the immune response to sciatic nerve injury identifies efferocytosis as a key mechanism of nerve debridement. eLife (2020) 9:e60223. doi: 10.7554/eLife.60223 33263277 PMC7735761

[32] Arai K Harada Y Tomiyama H Michishita M Kanno N Yogo T. Evaluation of the survival of bone marrow-derived mononuclear cells and the growth factors produced upon intramedullary transplantation in rat models of acute spinal cord injury. Res Vet Sci (2016) 107:88–94. doi: 10.1016/j.rvsc.2016.05.011 27473980

[33] Kim SJ Ko WK Jo MJ Arai Y Choi H Kumar H. Anti-inflammatory effect of Tauroursodeoxycholic acid in RAW 264.7 macrophages, Bone marrow-derived macrophages, BV2 microglial cells, and spinal cord injury. Sci Rep (2018) 8:3176. doi: 10.1038/s41598-018-21621-5 29453346 PMC5816629

[34] Norden DM Faw TD McKim DB Deibert RJ Fisher LC Sheridan JF. Bone marrow-derived monocytes drive the inflammatory microenvironment in local and remote regions after thoracic spinal cord injury. J Neurotrauma (2019) 36:937–49. doi: 10.1089/neu.2018.5806 PMC6484351 30014767

[35] Garcia-Bonilla L Brea D Benakis C Lane DA Murphy M Moore J. Endogenous protection from ischemic brain injury by preconditioned monocytes. J Neurosci (2018) 38:6722–36. doi: 10.1523/JNEUROSCI.0324-18.2018 PMC6067076 29946039

[36] Bauer C le Saux O Pomozi V Aherrahrou R Kriesen R Stolting S. Etidronate prevents dystrophic cardiac calcification by inhibiting macrophage aggregation. Sci Rep (2018) 8:5812. doi: 10.1038/s41598-018-24228-y 29643466 PMC5895639

[37] Chao H-H Li L Gao X Wang C Yue W. CXCL12 expression in aborted mouse uteri induced by IFN-γ: Potential anti-inflammatory effect involves in endometrial restoration after abortion in mice. Gene (2019) 700:38–46. doi: 10.1016/j.gene.2019.02.089 30898705

[38] Yu ZY Chen DW Tan CR Zeng GH He CY Wang J. Physiological clearance of Abeta by spleen and splenectomy aggravates Alzheimer-type pathogenesis. Aging Cell (2022) 21:e13533. doi: 10.1111/acel.13533 34939734 PMC8761003

[39] Li Y Li X Chen X Sun X Liu X Wang G. Qishen Granule (QSG) Inhibits Monocytes Released From the Spleen and Protect Myocardial Function via the TLR4-MyD88-NF-kappaB p65 Pathway in Heart Failure Mice. Front Pharmacol (2022) 13:850187. doi: 10.3389/fphar.2022.850187 35370707 PMC8964526

[40] Zhang K Lu WC Zhang M Zhang Q Xian PP Liu FF. Reducing host aldose reductase activity promotes neuronal differentiation of transplanted neural stem cells at spinal cord injury sites and facilitates locomotion recovery. Neural Regener Res (2022) 17:1814–20. doi: 10.4103/1673-5374.330624 PMC8820702 35017443

[41] Pinho AG Cibrao JR Lima R Gomes ED Serra SC Lentilhas-Graca J. Immunomodulatory and regenerative effects of the full and fractioned adipose tissue derived stem cells secretome in spinal cord injury. Exp Neurol (2022) 351:113989. doi: 10.1016/j.expneurol.2022.113989 35065953

[42] Bao F Bailey CS Gurr KR Bailey SI Rosas-Arellano MP Dekaban GA. Increased oxidative activity in human blood neutrophils and monocytes after spinal cord injury. Exp Neurol (2009) 215:308–16. doi: 10.1016/j.expneurol.2008.10.022 19056384

[43] Gris D Hamilton EF Weaver LC. The systemic inflammatory response after spinal cord injury damages lungs and kidneys. Exp Neurol (2008) 211:259–70. doi: 10.1016/j.expneurol.2008.01.033 18384773

[44] Aimaletdinov AM Gomzikova MO. Tracking of extracellular vesicles’ Biodistribution: new methods and approaches. Int J Mol Sci (2022) 23(19):11312. doi: 10.3390/ijms231911312 36232613 PMC9569979

[45] Driedonks T Jiang L Carlson B Han Z Liu G Queen SE. Pharmacokinetics and biodistribution of extracellular vesicles administered intravenously and intranasally to Macaca nemestrina. J extracellular Biol (2022) 1(10):e59. doi: 10.1002/jex2.59 36591537 PMC9799283

[46] Wiklander OPB Nordin JZ O’Loughlin A Gustafsson Y Corso G Mäger I. Extracellular vesicle in vivo biodistribution is determined by cell source, route of administration and targeting. (2015) 4:26316. doi: 10.3402/jev.v4.26316 PMC4405624 25899407

[47] Anderson KD. Targeting recovery: priorities of the spinal cord-injured population. J Neurotrauma (2004) 21:1371–83. doi: 10.1089/neu.2004.21.1371 15672628

[48] Freria CM Brennan FH Sweet DR Guan Z Hall JC Kigerl KA. Serial systemic injections of endotoxin (LPS) elicit neuroprotective spinal cord microglia through IL-1-dependent cross talk with endothelial cells. J Neurosci (2020) 40:9103. doi: 10.1523/JNEUROSCI.0131-20.2020 33051350 PMC7672996

[49] Narita M Kuzumaki N Narita M Kaneko C Hareyama N Miyatake M. Chronic pain-induced emotional dysfunction is associated with astrogliosis due to cortical δ-opioid receptor dysfunction. (2006) 97:1369–78. doi: 10.1111/j.1471-4159.2006.03824.x 16696849

[50] Hains BC Saab CY Klein JP Craner MJ Waxman SG. Altered sodium channel expression in second-order spinal sensory neurons contributes to pain after peripheral nerve injury. (2004) 24:4832–9. doi: 10.1523/JNEUROSCI.0300-04.2004% PMC6729453 15152043

[51] Tansley S Uttam S Ureña Guzmán A Yaqubi M Pacis A Parisien M. Single-cell RNA sequencing reveals time- and sex-specific responses of mouse spinal cord microglia to peripheral nerve injury and links ApoE to chronic pain. Nat Commun (2022) 13:843. doi: 10.1038/s41467-022-28473-8 35149686 PMC8837774

[52] Schomberg D Olson JK. Immune responses of microglia in the spinal cord: Contribution to pain states. Exp Neurol (2012) 234:262–70. doi: 10.1016/j.expneurol.2011.12.021 22226600

[53] Szklarczyk D Gable AL Nastou KC Lyon D Kirsch R Pyysalo S. The STRING database in 2021: customizable protein-protein networks, and functional characterization of user-uploaded gene/measurement sets. Nucleic Acids Res (2021) 49:D605–12. doi: 10.1093/nar/gkaa1074 PMC7779004 33237311

[54] Liu NK Zhang YP Han S Pei J Xu LY Lu PH. Annexin A1 reduces inflammatory reaction and tissue damage through inhibition of phospholipase A2 activation in adult rats following spinal cord injury. J neuropathology Exp Neurol (2007) 66:932–43. doi: 10.1097/nen.0b013e3181567d59 17917587

[55] Chalif JI Martínez-Silva ML Pagiazitis JG Murray AJ Mentis GZ. Control of mammalian locomotion by ventral spinocerebellar tract neurons. Cell (2022) 185:328–344.e326. doi: 10.1016/j.cell.2021.12.014 35063074 PMC8852337

[56] Lei Y Perez MA. Cerebellar contribution to sensorimotor adaptation deficits in humans with spinal cord injury. Sci Rep (2021) 11:2507. doi: 10.1038/s41598-020-77543-8 33510183 PMC7843630

[57] Formento E Minassian K Wagner F Mignardot JB Le Goff-Mignardot CG Rowald A. Electrical spinal cord stimulation must preserve proprioception to enable locomotion in humans with spinal cord injury. Nat Neurosci (2018) 21:1728–41. doi: 10.1038/s41593-018-0262-6 PMC6268129 30382196

[58] Matson KJE Russ DE Kathe C Hua I Maric D Ding Y. Single cell atlas of spinal cord injury in mice reveals a pro-regenerative signature in spinocerebellar neurons. Nat Commun (2022) 13:5628. doi: 10.1038/s41467-022-33184-1 36163250 PMC9513082

[59] Morris R Whishaw IQ. A proposal for a rat model of spinal cord injury featuring the rubrospinal tract and its contributions to locomotion and skilled hand movement. Front Neurosci (2016) 10:5. doi: 10.3389/fnins.2016.00005 26858587 PMC4728831

[60] van den Brand R Heutschi J Barraud Q DiGiovanna J Bartholdi K Huerlimann M. Restoring voluntary control of locomotion after paralyzing spinal cord injury. Science (2012) 336:1182–5. doi: 10.1126/science.1217416 22654062

[61] Borton D Bonizzato M Beauparlant J DiGiovanna J Moraud EM Wenger N. Corticospinal neuroprostheses to restore locomotion after spinal cord injury. Neurosci Res (2014) 78:21–9. doi: 10.1016/j.neures.2013.10.001 24135130

[62] Jo HJ Richardson MSA Oudega M Perez MA. Paired corticospinal-motoneuronal stimulation and exercise after spinal cord injury. J spinal cord Med (2021) 44:S23–s27. doi: 10.1080/10790268.2021.1970908 34779722 PMC8604481

[63] Jo HJ Perez MA. Corticospinal-motor neuronal plasticity promotes exercise-mediated recovery in humans with spinal cord injury. Brain (2020) 143:1368–82. doi: 10.1093/brain/awaa052 PMC7534104 32355959

[64] Wang C Zhang L Ndong JC Hettinghouse A Sun G Chen C. Progranulin deficiency exacerbates spinal cord injury by promoting neuroinflammation and cell apoptosis in mice. J Neuroinflamm (2019) 16:238. doi: 10.1186/s12974-019-1630-1 PMC6882111 31775776

[65] Shi Q Wu Y Zhang B Wu S Wang X Lin F. Progranulin promotes functional recovery in rats with acute spinal cord injury via autophagy-induced anti-inflammatory microglial polarization. Mol Neurobiol (2022) 59:4304–14. doi: 10.1007/s12035-022-02836-0 35505051

[66] Kuse Y Tsuruma K Mizoguchi T Shimazawa M Hara H. Progranulin deficiency causes the retinal ganglion cell loss during development. Sci Rep (2017) 7:1679. doi: 10.1038/s41598-017-01933-8 28490764 PMC5431873

[67] Menzel L Kleber L Friedrich C Hummel R Dangel L Winter J. Progranulin protects against exaggerated axonal injury and astrogliosis following traumatic brain injury. Glia (2017) 65:278–92. doi: 10.1002/glia.23091 27778404

[68] Liddelow SA Guttenplan KA Clarke LE Bennett FC Bohlen CJ Schirmer L. Neurotoxic reactive astrocytes are induced by activated microglia. Nature (2017) 541:481–7. doi: 10.1038/nature21029 PMC5404890 28099414

[69] Jha MK Jo M Kim J-H Suk K. Microglia-astrocyte crosstalk: an intimate molecular conversation. (2019) 25:227–40. doi: 10.1177/1073858418783959 29931997

[70] Brennan FH Li Y Wang C Ma A Guo Q Li Y. Microglia coordinate cellular interactions during spinal cord repair in mice. Nat Commun (2022) 13:4096. doi: 10.1038/s41467-022-31797-0 35835751 PMC9283484

[71] Lv C Zhang T Li K Gao K. Bone marrow mesenchymal stem cells improve spinal function of spinal cord injury in rats via TGF-beta/Smads signaling pathway. Exp Ther Med (2020) 19:3657–63. doi: 10.3892/etm.2020.8640 PMC7185179 32346429

[72] Pan D Yang F Zhu S Li Y Ning G Feng S. Inhibition of TGF-beta repairs spinal cord injury by attenuating EphrinB2 expressing through inducing miR-484 from fibroblast. Cell Death Discovery (2021) 7:319. doi: 10.1038/s41420-021-00705-8 34711831 PMC8553751

[73] Nakazaki M Morita T Lankford KL Askenase PW Kocsis JD. Small extracellular vesicles released by infused mesenchymal stromal cells target M2 macrophages and promote TGF-beta upregulation, microvascular stabilization and functional recovery in a rodent model of severe spinal cord injury. J Extracell Vesicles (2021) 10:e12137. doi: 10.1002/jev2.12137 34478241 PMC8408371

[74] Tyor WR Avgeropoulos N Ohlandt G Hogan EL. Treatment of spinal cord impact injury in the rat with transforming growth factor-beta. J Neurol Sci (2002) 200:33–41. doi: 10.1016/s0022-510x(02)00113-2 12127673

[75] Xu Y He X Wang Y Jian J Peng X Zhou L. 5-Fluorouracil reduces the fibrotic scar via inhibiting matrix metalloproteinase 9 and stabilizing microtubules after spinal cord injury. CNS Neurosci Ther (2022) 28:2011–23. doi: 10.1111/cns.13930 PMC9627390 35918897

[76] Li Y He X Kawaguchi R Zhang Y Wang Q Monavarfeshani A. Microglia-organized scar-free spinal cord repair in neonatal mice. Nature (2020) 587:613–8. doi: 10.1038/s41586-020-2795-6 PMC7704837 33029008

[77] Göritz C Dias DO Tomilin N Barbacid M Shupliakov O Frisén J. A pericyte origin of spinal cord scar tissue. Science (2011) 333:238–42. doi: 10.1126/science.1203165 21737741

[78] Dias DO Kim H Holl D Werne Solnestam B Lundeberg J Carlén M. Reducing pericyte-derived scarring promotes recovery after spinal cord injury. Cell (2018) 173:153–165.e122. doi: 10.1016/j.cell.2018.02.004 29502968 PMC5871719

[79] Narang A Zheng B. To scar or not to scar. Trends Mol Med (2018) 24:522–4. doi: 10.1016/j.molmed.2018.04.007 PMC5975241 29729835

[80] Santini MP Malide D Hoffman G Pandey G D'Escamard V Nomura-Kitabayashi A. Tissue-resident PDGFRα(+) progenitor cells contribute to fibrosis versus healing in a context- and spatiotemporally dependent manner. Cell Rep (2020) 30:555–570.e557. doi: 10.1016/j.celrep.2019.12.045 31940496 PMC7030884

[81] Moutal A White KA Chefdeville A Laufmann RN Vitiello PF Feinstein D. Dysregulation of CRMP2 post-translational modifications drive its pathological functions. Mol Neurobiol (2019) 56:6736–55. doi: 10.1007/s12035-019-1568-4 PMC6728212 30915713

[82] Kondo S Takahashi K Kinoshita Y Nagai J Wakatsuki S Araki T. Genetic inhibition of CRMP2 phosphorylation at serine 522 promotes axonal regeneration after optic nerve injury. Sci Rep (2019) 9:7188. doi: 10.1038/s41598-019-43658-w 31076621 PMC6510754

[83] Liz MA Mar FM Santos TE Pimentel HI Marques AM Morgado MM. Neuronal deletion of GSK3beta increases microtubule speed in the growth cone and enhances axon regeneration via CRMP-2 and independently of MAP1B and CLASP2. BMC Biol (2014) 12:47. doi: 10.1186/1741-7007-12-47 24923837 PMC4229956

[84] Gogel S Lange S Leung KY Greene ND Ferretti P. Post-translational regulation of Crmp in developing and regenerating chick spinal cord. Dev Neurobiol (2010) 70:456–71. doi: 10.1002/dneu.20789 20162635

[85] Tsuchida M Nakamachi T Sugiyama K Tsuchikawa D Watanabe J Hori M. PACAP stimulates functional recovery after spinal cord injury through axonal regeneration. J Mol neuroscience: MN (2014) 54:380–7. doi: 10.1007/s12031-014-0338-z 25074795

[86] Kase Y Sato T Okano Y Okano H. The GADD45G/p38 MAPK/CDC25B signaling pathway enhances neurite outgrowth by promoting microtubule polymerization. iScience (2022) 25:104089. doi: 10.1016/j.isci.2022.104089 35497000 PMC9042895

