# Supplementary material for: The secretome of macrophages has a differential impact on spinal cord injury recovery according to the polarization protocol
Source: Front Immunol. 2024 Feb 20;15:1354479. doi: 10.3389/fimmu.2024.1354479 (PMC10912310; doi:10.3389/fimmu.2024.1354479)
Supplement: Supplementary Figure 1 — Splenic macrophages characterization. (A) MCS-F is essential for the survival and proliferation and differentiation of splenic monocytes into macrophages. (B) After 24h of polarization with the pro-inflammatory molecules LPS and INF-γ, 89% of the macrophages expressed iNOS. (C) A total of 487 proteins identified, 81 were exclusive to the secretome of M(INF-γ+LPS) macrophages, 35 to M(IL-4+IL-13), and 90 to M(IL-10+TGF-β1). (D) Using the PANTHER tool was possible to identified metabolite interconversion enzymes (dark blue) as a common protein class between the different macrophage populations, but as can be observed by the pie charts, the protein class and the percentage of proteins in different classes varied considerably among each cell phenotype. Anti-CD11b antibody was used to identify macrophages (green), anti-iNOS antibody was used to confirm the polarization (red) and nuclei was counterstained with DAPI (blue). Scale bar =50 µm. 2 independent experiments were performed for the in vitro data and 1 independent experiment for proteomics analysis. [file Presentation_1.pdf]

Supplementary Material

The Secretome of Macrophages Has a Differential Impact on Spinal Cord Injury Recovery According to the Polarization Protocol

José Lentilhas-Graça[1,2,3], Diogo J. Santos[1,2#], João Afonso[1,2#], Andreia Monteiro[1,2], Andreia G. Pinho[1,2], Vera Mendes[3], Marta S. Dias[3,4], Eduardo D. Gomes[1,2], Rui Lima[1,2], Luís S. Fernandes[1,2], Fernando Fernandes-Amorim[1,2], Inês M. Pereira[1,2], Nídia de Sousa[1,2], Jorge R. Cibrão[1,2], Aline M. Fernandes[1,2], Sofia C. Serra[1,2], Luís A. Rocha[1,2], Jonas Campos[1,2], Tiffany S. Pinho[1,2], Susana Monteiro[1,2], Bruno Manadas[3], António J. Salgado[1,2], Ramiro D. Almeida[3,4], Nuno A. Silva[1,2,*]

Supplementary Figures

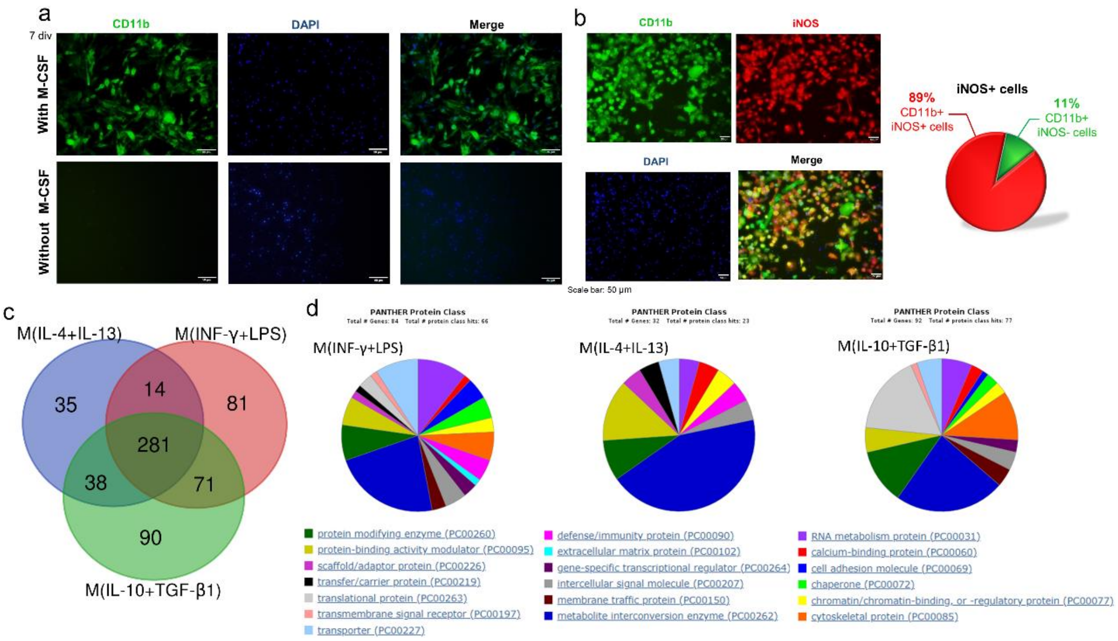

Supplementary Figure 1: Splenic macrophages characterization. **a**) MCS-F is essential for the survival and proliferation and differentiation of splenic monocytes into macrophages. **b**) After 24h of polarization with the pro-inflammatory

molecules LPS and INF-γ, 89% of the macrophages expressed iNOS. **c**) A total of 487 proteins identified, 81 were exclusive to the secretome of M($_{\text{INF-γ+LPS}}$) macrophages, 35 to M($_{\text{IL-4+IL-13}}$), and 90 to M($_{\text{IL-10+TGF-β1}}$). **d**) Using the PANTHER tool was possible to identified metabolite interconversion enzymes (dark blue) as a common protein class between the different macrophage populations, but as can be observed by the pie charts, the protein class and the percentage of proteins in different classes varied considerably among each cell phenotype. Anti-CD11b antibody was used to identify macrophages (green), anti-iNOS antibody was used to confirm the polarization (red) and nuclei was counterstained with DAPI (blue). Scale bar =50 µm. 2 independent experiments were performed for the in vitro data and 1 independent experiment for proteomics analysis.

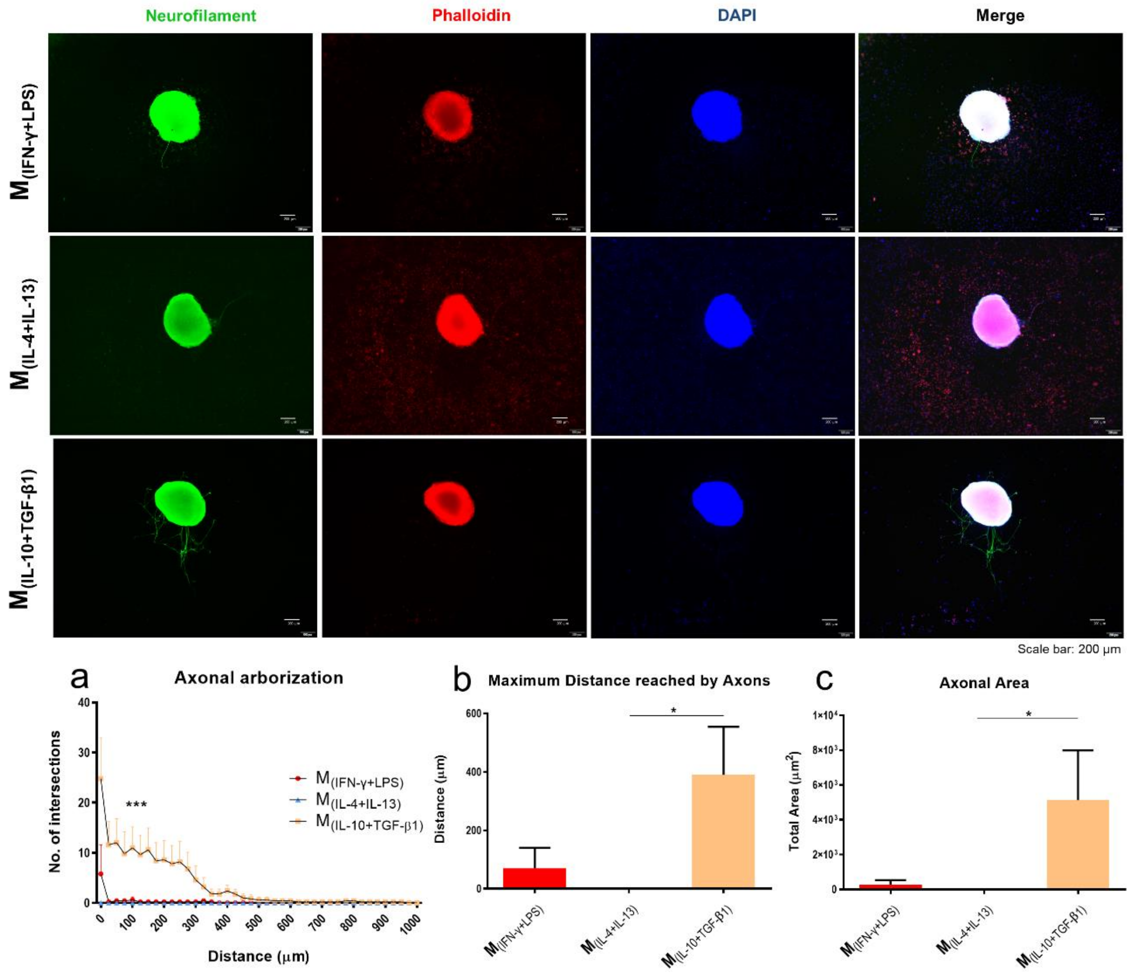

Supplementary Figure 2: Classical (M($_{\text{INF-γ+LPS}}$)) or alternative (M($_{\text{IL-4+IL-13}}$); M($_{\text{IL-10+TGF-β1}}$)) activated macrophages co-cultured with dorsal root ganglia (DRGs) in 2D. DRGs stained with Neurofilament (green), Macrophages and DRGs stained

with Phalloidin (red) and nuclei counterstained with DAPI (blue). **a**) DRGs co-cultured with $M_{(IL-10+TGF-\beta1)}$ macrophages had significantly higher axonal arborisation (3, 17 df, p<0.0001) and **b**) significantly longer axons (p=0.0247) than the than $M_{(IL-4+IL-13)}$. **c**) $M_{(IL-10+TGF-\beta1)}$ also had significant more axonal area (0.0240) than the $M_{(IL-4+IL-13)}$. Two way ANOVA followed by Tukey post-hoc test was used for axonal arborization analysis and Kruskal-Wallis test followed by Dunn's multiple comparisons test was used for longer distance and axonal area analysis. Data is presented as mean ± standard error (SEM). *- $p < 0.05$; ***- $p < 0.001$. Scale bar =200 µm; $M_{(INF-\gamma+LPS)}$ n=5; $M_{(IL-4+IL-13)}$ n=4; $M_{(IL-10+TGF-\beta1)}$ n=5. 2 independent experiments were performed.

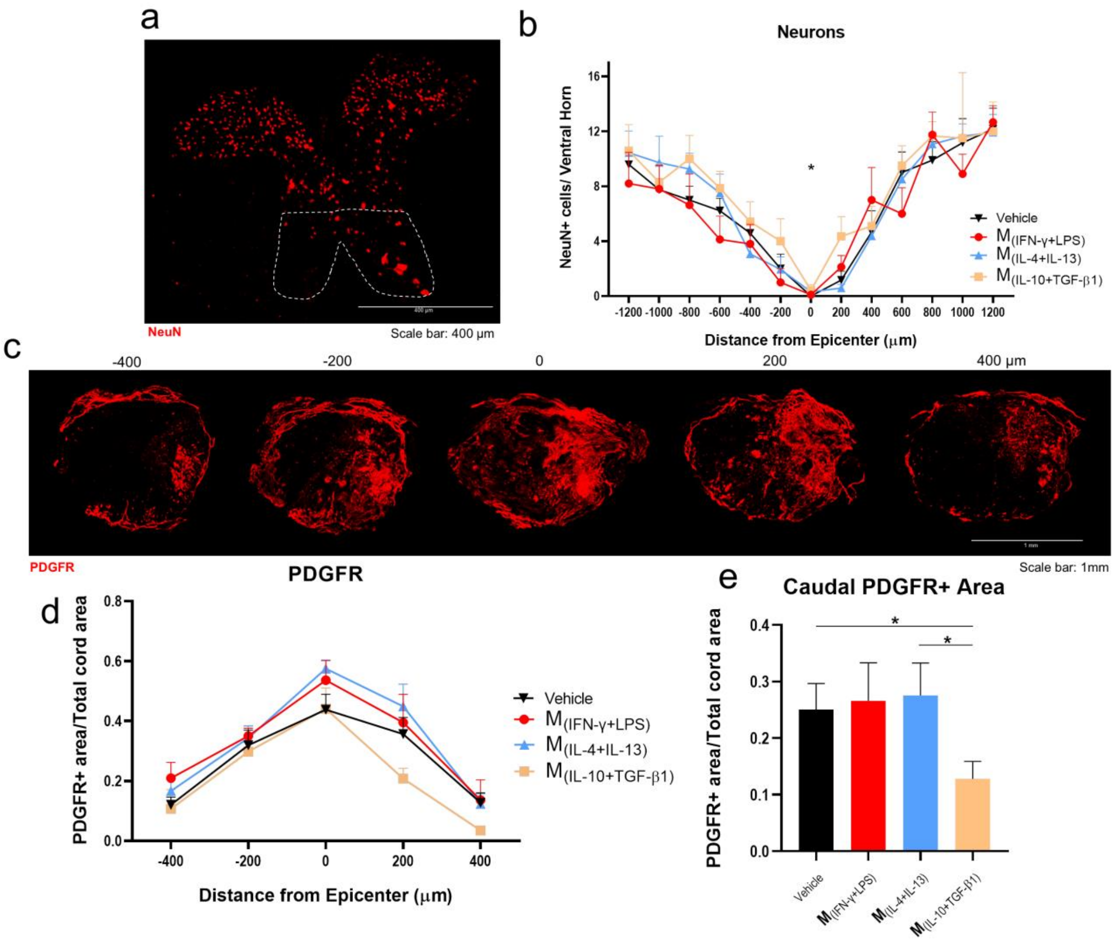

Supplementary Figure 3: Histological analysis of the spinal cord. **a**) Representative image of gray matter neurons from $M_{(IL-10+TGF-\beta1)}$-treated group, cell bodies were measured by counting the number of positive NeuN cells (red) in laminae VIII and IX of both ventral horns. **b**) Rostral-caudal analysis

demonstrated that the secretome derived from $M_{(IL-10+TGF-\beta 1)}$ cells significantly promoted neuronal survival at the ventral horns (3, 253 df, p= 0.0438) when compared with $M_{(IFN-\gamma+LPS)}$. **c**) Representative image of fibrotic scar from vehicle-treated group, anti-PDGFRβ antibody (red) was used to analyse fibrosis in the spinal cord. **d**) Although there are not significant differences in PDGFRβ+ total area between treated groups, **e**) rostral caudal analysis show that mice treated with $M_{(IL-10+TGF-\beta 1)}$-derived secretome had significantly less fibrosis caudally to the injury area (3, 49 df, p= 0.0370). ANOVA followed by the Tukey post-hoc test was used to analyse statistical differences. A total of 312 spinal cord slices were observed to analyse neuronal survival and 134 slices (53 for the caudal calculation) for fibrosis. Data is presented as mean ± standard error (SEM). *- p < 0.05. Vehicle n=7; $M_{(INF-\gamma+LPS)}$ n=6; $M_{(IL-4+IL-13)}$ n=8; $M_{(IL-10+TGF-\beta 1)}$ n=6. 1 independent experiment was performed.

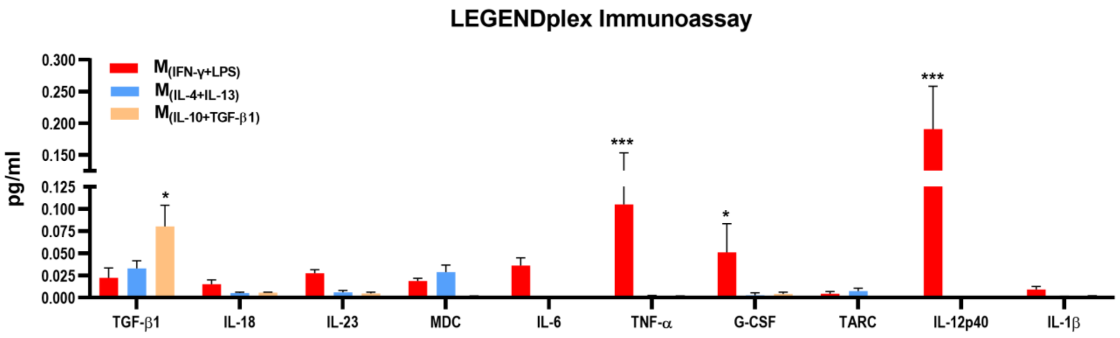

Supplementary Figure 4: LEGENDplex immunoassay. The pro-inflammatory cytokines TNF-α, G-CSF and IL12p40 were significantly concentrated on the secretome of $M_{(INF-\gamma+LPS)}$. The cytokine/hormone G-CSF was also significantly concentrated on the $M_{(INF-\gamma+LPS)}$-derived secretome. TGF-β1, a cytokine with anti-inflammatory properties was significantly concentrated in the $M_{(IL-10+TGF-\beta 1)}$-derived secretome. Data was analysed using the two-way ANOVA (2, 130 df, p<0.0001) followed by the Tukey's multiple comparisons test. Data is presented as mean ± standard error (SEM). *- p < 0.05; ***- p < 0.001, , $M_{(IFN-\gamma+LPS)}$ n=4; $M_{(IL-4+IL-13)}$ n=6; $M_{(IL-10+TGF-\beta 1)}$ n=6. Concentration values plotted in the graph were divided by 10 to account for the concentration step performed before the analysis. The values for CXCL-1, IL-12p70, and IL-10 were below the limit of detection and

were consequently excluded from the analysis. 1 independent experiment was performed.

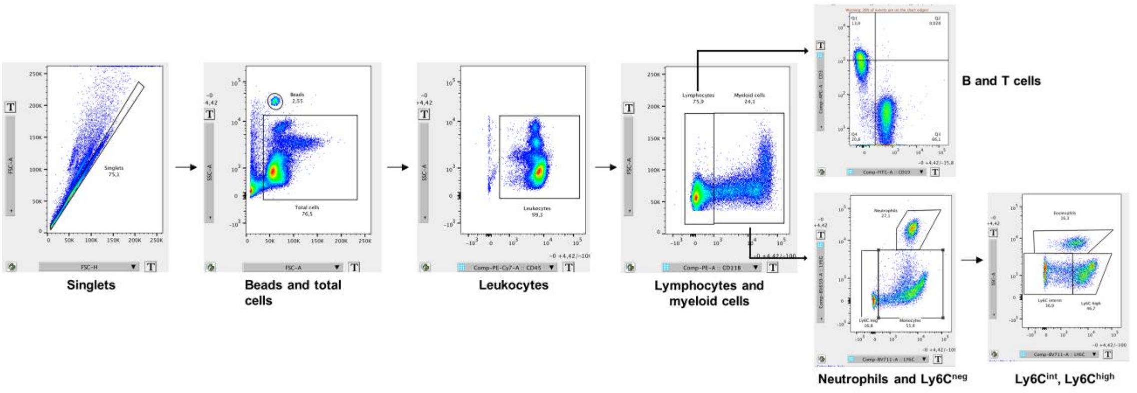

Supplementary Figure 5: Gating strategy used for flow cytometry analysis of mice blood cells. Doublets were excluded by FSC-A vs FSC-H scatter. Blood total cells were gated by SSC-A vs FSC-A scatter. Leukocytes were gated by CD45+ cells and on this population lymphocytes and myeloid cells were distinguished by CD11b expression. In lymphocytes population, CD3+CD19- cells were defined as T cells and CD3-CD19+ cells were defined as B cells. In myeloid cell population, Ly6G vs Ly6C allowed the selection of neutrophils (Ly6G+Ly6C+) and monocytes (Ly6G-Ly6C+). The selection of eosinophils vs monocytes Ly6C intermediate vs monocytes Ly6C high was made based on Ly6C vs SSC-A gating.

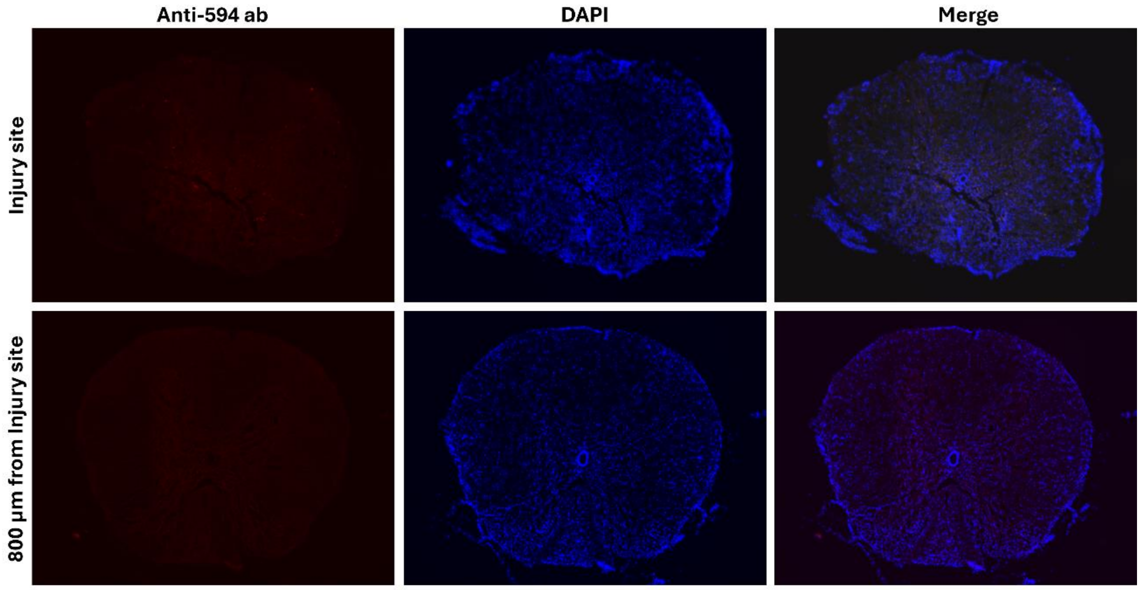

Supplementary Figure 6: Negative control fluorescence images of the Alexa Fluor 594 goat anti-rabbit antibody (red) both at the injury site and 800μm from the injury site. DAPI (blue) was used as structural marker.
